# Polymeric Nanoparticles for Targeted Lung Cancer Treatment: Review and Perspectives

**DOI:** 10.3390/pharmaceutics17091091

**Published:** 2025-08-22

**Authors:** Devesh U. Kapoor, Sonam M. Gandhi, Sambhavi Swarn, Basant Lal, Bhupendra G. Prajapati, Supang Khondee, Supachoke Mangmool, Sudarshan Singh, Chuda Chittasupho

**Affiliations:** 1 Department of Pharmaceutics, Dr. Dayaram Patel Pharmacy College, Bardoli 394601, Gujarat, India; dev7200@gmail.com; 2 Department of Pharmaceutics, Shree Dhanvantary Pharmacy College, Dist-Kim, Olpad 394110, Gujarat, India; shena.saloni@gmail.com (S.M.G.); sambhaviamresh24@gmail.com (S.S.); 3 PQE Australia Pvt. Ltd., Melbourne, VIC 300, Australia; basant.tyagi63601@gmail.com; 4 Department of Pharmaceutics, Parul Institute of Pharmacy, Faculty of Pharmacy, Parul University, Waghodia, Vadodara 391760, Gujarat, India; bhupendra.prajapati40731@paruluniversity.ac.in; 5 Faculty of Pharmacy, Silpakorn University, Nakhon Pathom 73000, Thailand; 6 Centre for Research Impact and Outcome, Chitkara College of Pharmacy, Chitkara University, Rajpura 140401, Punjab, India; 7 School of Pharmaceutical Sciences, University of Phayao Maek, Muaeng, Phayao 56000, Thailand; supang.kh@up.ac.th; 8 Faculty of Pharmacy, Chiang Mai University, Chiang Mai 50200, Thailand; supachoke.man@cmu.ac.th; 9 Office of Research Administration, Chiang Mai University, Chiang Mai 50200, Thailand

**Keywords:** polymeric nanoparticles, lung cancer, anticancer, molecular pathways, targeted therapy

## Abstract

Lung cancer remains a foremost cause of cancer-related impermanence globally, demanding innovative and effective therapeutic strategies. Polymeric nanoparticles (NPs) have turned up as a promising transport system for drugs due to their biodegradability, biocompatibility, and capability to provide controlled and targeted release of therapeutic agents. This review offers a thorough examination of different polymeric NP platforms, such as chitosan, gelatin, alginate, poly (lactic acid), and polycaprolactone, highlighting their mechanisms, formulations, and applications in the treatment of lung cancer. These NPs facilitate the delivery of chemotherapeutic agents, gene therapies, and immune modulators, with enhanced bioavailability and reduced systemic toxicity. Additionally, advanced formulations such as ligand-conjugated, stimuli-responsive, and multifunctional NPs demonstrate improved tumor-specific accumulation and cellular uptake. The review also discusses quantum dots, magnetic and lipid-based NPs, and green-synthesized metallic polymeric hybrids, emphasizing their potential in theranostics and combination therapies. Preclinical studies show promising results, yet clinical translation faces challenges; for example, large-scale production, long-term toxicity, and regulatory hurdles. Overall, polymeric NPs represent a powerful platform for advancing personalized lung cancer therapy, with future prospects rooted in multifunctional, targeted, and patient-specific nanomedicine.

## 1. Introduction

Lung cancer continues to be the most fatal cancer globally, accounting for approximately 1.8 million deaths in 2020, alongside 2.2 million newly diagnosed cases [1,2]. Regions such as Europe, Eastern Asia, and Southeast Asia report the highest prevalence, with the use of tobacco identified as the key risk factor, contributing to nearly 85% of all cases [3]. However, non-smoking-related lung cancer, often linked to genetic mutations (e.g., epidermal growth factor receptor (EGFR), anaplastic lymphoma kinase [4] receptors) and environmental exposures (e.g., radon, air pollution), accounts for 10–25% of cases globally [5,6,7,8,9].

**Figure 1:**
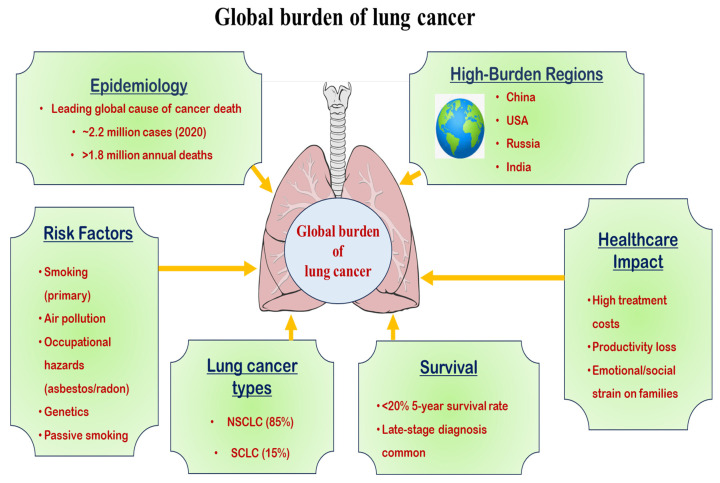
Diagrammatic representation of the global burden of lung cancer.

Age-standardized incidence rates are higher in men, though increasing trends among women, particularly in high-income countries, are concerning [10]. Socioeconomic disparities also influence outcomes, with lower survival rates due to delayed detection and inadequate treatment availability, which low- and middle-income nations face [1,11,12]. Advances in early detection (e.g., low-dose computerized tomography (CT) screening) and targeted therapies have improved survival in high-resource settings, yet global disparities persist (National Lung Screening Trial Research Team 2011). Figure 1 illustrates the trends, disparities, and emerging risks of lung cancer globally.

**Figure 2:**
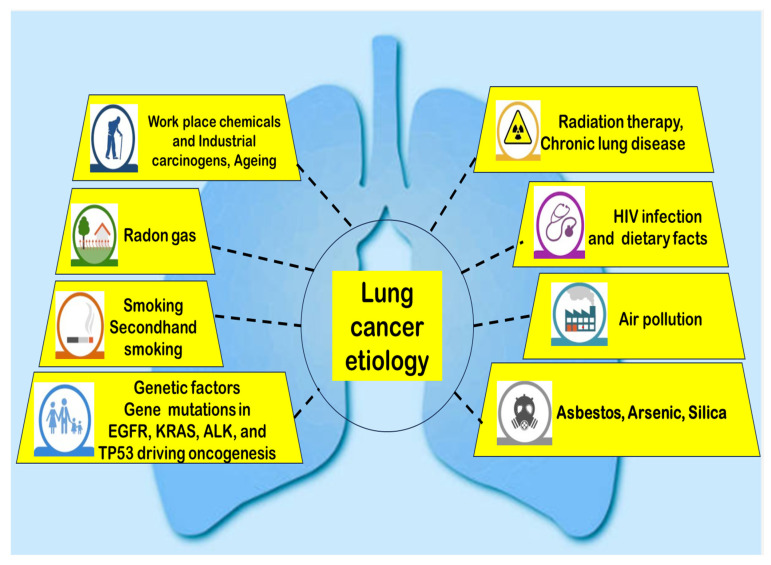
Illustration of the etiology of lung cancer.

Lung cancer arises from a complicated interaction of behavioral, environmental, and genetic factors. Tobacco smoking remains the primary cause, contributing to 80–90% of cases, with carcinogens like polycyclic aromatic hydrocarbons and nitrosamines inducing DNA mutations [1]. However, 15–20% of lung cancers occur in people who do not have a smoking habit, with risk factors including radon gas, secondhand smoke, air pollution (e.g., PM_2_._5_), and occupational exposures (asbestos, arsenic, silica) [13] (Figure 2).

Genetic susceptibility plays a crucial role, with mutations in EGFR, KRAS (Kirsten rat sarcoma virus), ALK, and Tumor suppressor (TP) 53 driving oncogenesis, particularly in non-smokers [14]. Additionally, chronic inflammation from conditions like chronic obstructive pulmonary disease (COPD) and pulmonary fibrosis elevates lung cancer risk [15]. Emerging evidence suggests dietary factors (low fruit/vegetable intake) and hormonal influences (estrogen signaling) may contribute, though further research is needed [15]. In recent years, electronic cigarette (e-cigarette) use has raised concerns due to potential carcinogenic effects, though long-term studies are still lacking [15]. Understanding these multifactorial causes is essential for preventive strategies and personalized treatment approaches.

Lung cancer pathogenesis involves the dysregulation of key molecular pathways that drive tumor initiation, progression, and metastasis. The EGFR/RAS/RAF/MEK/ERK and PI3K/AKT/mTOR pathways are frequently activated, promoting cell proliferation and survival [16]. Mutations in EGFR, KRAS, and BRAF are common in non-small cell lung cancer (NSCLC), while ALK, ROS1, and RET rearrangements define distinct molecular subtypes [15]. In small-cell lung cancer (SCLC), TP53 and RB1 inactivation are nearly universal, leading to unchecked cell cycle progression [17]. Epigenetic alterations, such as DNA methylation and histone modifications, further contribute to oncogenic signaling [18]. The immune checkpoint pathways (PD-1/PD-L1, CTLA-4) play a critical role in tumor immune evasion, making them key targets for immunotherapy [18]. Emerging research highlights the role of tumor microenvironment [19] components, including cancer-associated fibroblasts and immune cells, in modulating therapeutic resistance [20]. Additionally, WNT/β-catenin and Hippo-YAP/TAZ signaling pathways are implicated in lung cancer stemness and metastasis [20].

Polymeric nanoparticle systems can be strategically designed not only to deliver therapeutic agents but also to actively remodel the tumor microenvironment [19] to overcome resistance mechanisms. Beyond stimuli-responsive release, PNPs can be engineered to co-deliver immunomodulators, such as checkpoint inhibitors or cytokines that reprogram immunosuppressive TMEs into immunostimulatory ones, enhancing antitumor immunity. Surface functionalization with ligands targeting cancer-associated fibroblasts or tumor-associated macrophages can disrupt stromal barriers, reduce extracellular matrix (ECM) density, and improve drug penetration [21]. Incorporating enzymes like hyaluronidase or collagenase within PNPs enables localized ECM degradation, lowering interstitial fluid pressure and enhancing vascular perfusion. Polymeric NPs can also deliver agents that inhibit pro-survival signaling pathways (e.g., STAT3, HIF-1α) or modulate hypoxia by carrying oxygen-generating compounds, thereby sensitizing tumors to chemotherapy or radiotherapy [22]. Additionally, NPs can be designed to release reactive oxygen species modulators to disrupt redox balance, making cancer cells more vulnerable to therapy. By integrating these TME-modulating capabilities with precise drug delivery, polymeric NPs offer a multifunctional approach to simultaneously overcome physical, biochemical, and immunological resistance barriers, ultimately improving therapeutic efficacy in lung cancer management [23].

## 2. Targeted Therapies in Lung Cancer

Current progress in nanotechnology has led to the development of advanced drug delivery methods, enhancing the effectiveness of cancer therapy while reducing harmful adverse effects [24,25,26,27]. Among these, liposomal formulations remain clinically dominant due to their biocompatibility and ability to encapsulate both hydrophilic and hydrophobic drugs [28]. Modern liposomes incorporate surface modifications, such as PEGylation, to extend circulation times and antibody conjugation for active tumor targeting.

Polymeric NPs, particularly those using biodegradable poly(lactic-co-glycolic acid) (PLGA), demonstrate excellent controlled-release properties through tunable polymer degradation rates [29,30]. Recent innovations include stimuli-responsive designs that release payloads specifically in acidic tumor microenvironments [31,32]. Dendrimers offer unparalleled structural precision for high drug-loading capacity and functionalization, though toxicity concerns require further investigation [33].

Inorganic NPs, such as gold nanostructures, provide dual diagnostic and therapeutic capabilities through photothermal ablation and imaging contrast enhancement [34]. Meanwhile, natural exosome-based systems show promise for their innate biocompatibility and homing capabilities to specific tissues [35]. Current research focuses on hybrid systems combining multiple nanocarrier advantages while addressing manufacturing scalability and regulatory requirements for clinical translation.

However, translating polymeric NPs from bench to bedside in lung cancer therapy faces multifaceted challenges. Scalability remains a primary hurdle, as achieving reproducible size, drug loading, and surface characteristics during large-scale manufacturing is technically demanding and cost-intensive [36]. Regulatory considerations add complexity, with evolving guidelines for nanomedicines requiring exhaustive characterization, toxicological profiling, and demonstration of long-term safety. Safety evaluation is critical, given the potential for unforeseen immunogenicity, off-target accumulation, or toxicity from degradation products, particularly with repeated dosing [37]. Comprehensive in vitro and in vivo studies, alongside predictive models, are essential for risk assessment. Eventually, cost-effectiveness poses a barrier; despite promising therapeutic outcomes, high production and quality control costs can limit commercial viability and patient accessibility. Addressing these challenges will require standardized manufacturing protocols, robust regulatory frameworks, advanced safety analytics, and cost-reduction strategies to ensure clinical translation and broader adoption of PNP-based lung cancer therapeutics [38].

### Comparison Between Conventional and Novel Therapies Involved in the Management of Lung Cancer

Traditional approaches to managing lung carcinoma, such as surgical resection, systemic chemotherapy, and radiotherapy, have historically served as the primary treatment options. Nevertheless, these methods frequently exhibit poor precision, resulting in severe adverse effects and reduced effectiveness, particularly in late-stage disease [39]. Emerging treatment strategies, including molecularly targeted agents, offer promising alternatives. Therapy, immunotherapy, and nanotechnology-based drug delivery offer improved selectivity, reduced systemic toxicity, and enhanced therapeutic outcomes. Targeted therapies inhibit specific molecular pathways, while immunotherapy boosts the body’s immune response against cancer. Nanocarriers enhance drug accumulation at tumor sites [40]. Although novel drug delivery suppresses the disadvantages of conventional therapies, the mucociliary clearance is indeed a critical physiological barrier limiting the pulmonary residence time of inhalable therapeutics. In the formulation, the polymeric NPs were engineered with a muco-inert surface by incorporating PEGylated polymers, which reduce adhesive interactions with mucus glycoproteins, thereby enabling deeper penetration into the lung epithelium. Furthermore, the NPs possess a narrow size distribution (~200 nm) to avoid rapid entrapment in mucus mesh pores while facilitating alveolar deposition [41]. The hydrophilic polymer coating also minimizes opsonization and phagocytic uptake by alveolar macrophages, supporting prolonged drug retention. In addition, the controlled-release matrix of the polymer enables sustained drug liberation at the target site, countering clearance-driven loss. Collectively, these features synergistically mitigate mucociliary removal, enhance deep lung bioavailability, and ensure prolonged therapeutic levels at the tumor site, thereby maximizing the clinical potential of our inhalable polymeric NP system for localized lung cancer therapy [42]. Therefore, overall, novel approaches provide personalized and more effective management, especially for NSCLC, though they may still face challenges like resistance and high cost. A comparative data on conventional and novel therapies for lung cancer management is presented in Table 1.

## 3. Advanced Nanoparticle Platforms for Lung Cancer Theranostics and Targeted Therapy

### 3.1. Magnetic Nanoparticles

Magnetic NPs have emerged as efficient tools in lung cancer diagnosis and treatment because of their unique physicochemical properties. Superparamagnetic iron oxide NPs are extensively studied, enabling magnetic resonance imaging (MRI) enhancement for early tumor detection with high sensitivity [56]. Functionalized MNPs conjugated with targeting ligands (e.g., EGFR antibodies) improve tumor-specific drug delivery, reducing systemic toxicity [57].

In hyperthermia therapy, alternating magnetic fields heat Magnetic NPs to 42–46 °C, inducing selective cancer cell apoptosis while sparing healthy tissue [58]. Combined with chemotherapy, e.g., doxorubicin (DOX)-loaded MNPs, this approach enhances tumor penetration and treatment efficacy [57]. MNPs also facilitate gene therapy by delivering siRNA to silence oncogenes (e.g., KRAS) [59]. Recent advances include multifunctional MNPs integrating imaging, therapy, and real-time monitoring [60]. For example, superparamagnetic iron oxide NPs coated with PD-L1 inhibitors enable immunotherapy alongside MRI tracking [61]. Challenges remain in optimizing biocompatibility, biodistribution, and large-scale production [62].

### 3.2. Solid Lipid Nanoparticles

Solid lipid nanoparticles (SLNs) have gained attention as efficient drug transport systems for lung cancer due to their stability, biocompatibility, and ability to encapsulate both water-loving and hydrophobic drugs [63]. Composed of physiological lipids, SLNs increased tumor targeting while reducing the systemic toxicity [64]. Their small size (50–300 nm) facilitates passive accumulation in tumors via the enhanced permeability and retention (EPR) effect, improving drug bioavailability [64]. SLNs are particularly valuable for delivering chemotherapeutic agents (e.g., paclitaxel, docetaxel) with reduced side effects compared to conventional formulations [65]. Surface modification with ligands (e.g., folate, transferrin) enables active targeting of cancer cells overexpressing specific receptors [66]. Additionally, SLNs can co-deliver siRNA or miRNA to silence oncogenic pathways (e.g., EGFR, KRAS), enhancing therapeutic efficacy [67]. Recent studies highlight SLNs in combination therapy, such as co-loading chemotherapeutics with immunomodulators (e.g., PD-1 inhibitors) to synergize treatment [64]. SLNs also show promise in inhalable formulations, enabling localized lung delivery and bypassing first-pass metabolism [68].

### 3.3. Polymeric Reduced Green Metallic Nanoparticles

Polymeric reduced green metallic (PRGM) NPs represent a convergence of green chemistry and nanotechnology, offering an eco-friendly alternative, sustainable to conventional nanoparticle synthesis methods. These NPs are synthesized using biocompatible polymers and reducing agents derived from natural plant extracts, microbes, or other biotic resources, eliminating the need for hazardous chemicals [69]. PRGM NPs have emerged as a novel and sustainable approach in lung cancer therapy, integrating the principles of nanotechnology, green chemistry, and polymer science. These NPs are synthesized using either natural or synthetic polymeric materials as reducing agents, offering an eco-friendly alternative to conventional chemical methods [69]. The green synthesis minimizes toxicity while improving biocompatibility, essential traits for cancer therapeutics. Incorporating biopolymers such as chitosan, PLGA, or polyethylene glycol (PEG) into the nanoparticle matrix enhances stability, targeted delivery, and controlled drug release. Further, the variability in process of fabrication of NPs can be minimized through authenticated sourcing, purity, and standardized synthesis process in case of synthetic polymers and extraction protocols for natural gums. Furthermore, the process standardization includes fixed parameters (pH, temperature, isolation-to-precursor ratio) and quality control checkpoints to monitor precursor quality, extraction yield, and NPs characteristics. This approach enhances reproducibility, comparability, and therapeutic predictability while reducing batch-to-batch variability. PRGM NPs can be engineered to carry chemotherapeutic agents or imaging molecules, enabling simultaneous treatment and diagnosis (theranostics). Their surface can be functionalized for tumor-specific targeting, allowing for improved accumulation in cancerous lung tissues via the EPR effect or active targeting ligands.

In lung cancer, where drug resistance and systemic toxicity pose significant challenges, polymeric reduced green metallic NPs provide enhanced cellular uptake, reduced off-target effects, and the potential to bypass multidrug resistance pathways [70]. Additionally, their antioxidant and anti-inflammatory properties derived from the plant extracts used in synthesis can contribute to synergistic therapeutic effects. Overall, polymeric reduced green metallic NPs represent a promising and sustainable nanoplatform for advancing lung cancer treatment, offering high efficacy with minimized side effects and ecological impact [71]. Table 2 illustrates the comprehensive tabular summary of PRGM NPs in lung cancer management.

### 3.4. Quantum Dots

Quantum dots (QDs) are semiconductor nanocrystals, typically 2–10 nm in diameter, that exhibit unique size-dependent optical and electronic properties due to quantum confinement effects [76]. They possess high photostability, tunable emission wavelengths, and exceptional brightness, making them highly suitable for biomedical imaging, targeted drug delivery, and multifunctional theranostic applications [77]. Their surfaces can be functionalized with biomolecules, drugs, or polymers, enabling simultaneous diagnostic and therapeutic functions within a single nanoplatform.

Many QDs especially those with cadmium selenide (CdSe), cadmium telluride (CdTe), or lead-based cores pose significant heavy metal toxicity risks. Cadmium ions, released through core degradation under physiological conditions, can induce oxidative stress, mitochondrial damage, and DNA strand breaks, leading to mutagenesis and carcinogenesis. These effects are exacerbated by the small size and large surface area of QDs, which facilitate cellular uptake and interaction with biomolecules [77]. Chronic exposure or incomplete clearance may result in accumulation in organs such as the liver, spleen, kidneys, and brain, raising concerns over nephrotoxicity, hepatotoxicity, and neurotoxicity. Long-term persistence could also trigger chronic inflammation and immune dysregulation, potentially contributing to fibrosis or secondary malignancies. While surface coatings (e.g., PEG, silica, or polymer shells) and heavy-metal-free alternatives (e.g., carbon or indium phosphide QDs) can reduce immediate toxicity, their long-term safety profiles remain underexplored [78]. Comprehensive in vivo studies covering biodistribution, degradation pathways, clearance rates, and delayed toxic effects are essential before clinical translation. Without such evaluation, the full risk profile of QD-based lung cancer theranostics remains uncertain, particularly for repeated or high-dose applications [78].

QDs exhibit substantial promise in revolutionizing lung cancer theranostics through multifunctional capabilities. Research demonstrates that QDs functionalized with targeting ligands, such as EGFR antibodies or RGD peptides, significantly enhance the selective delivery of chemotherapeutic agents (e.g., DOX, cisplatin) to lung tumor cells in vitro and animal models, improving efficacy while minimizing off-target effects compared to free drugs [79]. Their exceptional optical properties are leveraged for superior imaging; specifically, near-infrared-II (NIR-II) window QDs enable deep-tissue, high-resolution tumor visualization, precise lymphatic mapping, and real-time monitoring of drug distribution and therapeutic response, surpassing conventional imaging agents in sensitivity and photostability [80]. The inherent theranostic potential of QDs is further exploited in integrated platforms where QDs co-loaded with drugs and photosensitizers facilitate combined chemotherapy and photodynamic therapy, generating reactive oxygen species (ROS) upon light activation for synergistic tumor cell killing, as confirmed in recent studies [81]. Addressing biocompatibility, significant research focuses on mitigating potential toxicity: surface engineering with dense PEG layers or biocompatible polymers reduces opsonization and prolongs circulation [82]. Furthermore, QDs are being explored as carriers for siRNA targeting oncogenes (e.g., survivin, KRAS) and for immune checkpoint inhibitors (e.g., anti-PD-L1), demonstrating enhanced gene silencing efficacy and potentiation of antitumor immunity in lung cancer models [83]. Despite compelling preclinical evidence for enhanced targeting, imaging, combination therapy, and improved safety profiles of novel compositions, the translation of QD-based systems necessitates rigorous large-scale toxicity assessments and scalable manufacturing solutions [84].

## 4. Application of Polymeric Nanoparticles in Lung Cancer Management

Polymeric nanoparticles are colloidal systems composed of synthetic or natural polymers, typically measuring between 10 and 1000 nm in size. These NPs are engineered to encapsulate, protect, and transport therapeutic agents to specific target areas, enabling controlled drug release and improved bioavailability. Due to their biocompatibility and tunable surface characteristics, they are extensively utilized in targeted drug delivery applications [85]. In lung cancer therapy, polymeric NPs significantly improve treatment effectiveness while lowering systemic toxicity. They facilitate the targeted delivery of chemotherapeutic drugs, immune checkpoint inhibitors, or gene therapies directly to tumor sites, enhancing drug concentration in cancerous tissues while sparing healthy lung cells [86]. Additionally, polymeric NPs can overcome biological barriers and release drugs in response to tumor-specific triggers, such as acidic pH or elevated enzyme levels. This targeted approach enhances therapeutic efficiency while reducing adverse effects often seen with traditional lung cancer treatments [87]. Different polymers exhibit distinct degradation mechanisms and by-products, influencing long-term safety in pulmonary delivery. Chitosan degrades enzymatically via lysozyme to glucosamine and N-acetylglucosamine, both naturally occurring metabolites with low toxicity [88]. Alginate undergoes depolymerization by alginate lyase to guluronic and mannuronic acid residues, which are generally well tolerated [89]. PLGA hydrolyzes into lactic and glycolic acid, entering the Krebs cycle, though rapid degradation in poorly ventilated lung regions may cause transient local acidity [90]. A comparative overview of key polymers, targeting approaches, and delivery routes for polymeric NPs in lung cancer therapy has been presented in Table 3.

Polymeric NPs can be engineered for multimodal theranostic applications in lung cancer by integrating various imaging agents. Magnetic resonance imaging labels, such as superparamagnetic iron oxide or gadolinium chelates, provide high soft-tissue contrast without ionizing radiation, though sensitivity is lower than nuclear techniques. Computed tomography (CT) contrast can be achieved by incorporating high-Z elements (gold, bismuth, iodine), enabling excellent spatial resolution for lung anatomy but at the cost of radiation exposure [100]. Positron emission tomography and single photon emission computed tomography use radio-labeled NPs for highly sensitive, quantitative molecular imaging, with PET offering higher resolution but shorter isotope half-lives. Optical imaging, particularly near-infrared modalities, enables real-time, high-resolution visualization for surgical guidance, although tissue penetration is limited in the thoracic region. Ultrasound and photoacoustic imaging employ NP-encapsulated acoustic or optical absorbers for real-time, radiation-free imaging, but pulmonary air spaces limit deep-tissue access. Hybrid PNP systems combining multiple imaging modalities can leverage complementary strengths for improved diagnosis, image-guided therapy, and treatment monitoring in lung cancer theranostics [101]. A summary on different imaging modalities integrated with polymeric nanoparticles for lung cancer theranostics is presented in Table 4.

### 4.1. Chitosan-Based Nanoparticles

Traditional chemotherapy methods face numerous challenges, such as rapid drug clearance, poor drug targeting, multidrug resistance, and limited therapeutic efficiency. Chitosan, a polymer naturally derived from chitin via deacetylation, comprises β-(1-4)-linked d-glucosamine and N-acetyl-*d*-glucosamine units [92]. Its favorable properties are biocompatibility, biodegradability, and mucoadhesiveness, which make it a suitable candidate for developing nanoparticulate drug delivery systems. Due to the presence of free amino groups, it is modifiable and allows functionalization according to specific therapeutic needs, specifically for lung cancer treatment. Recent advancements in chitosan NPs (CNPs) demonstrate their promising application in targeted drug delivery [107].

Pawar and Jaganathan illustrated the high loading capacity of glycol-modified CNPs for hepatitis B vaccines, supporting their ability in mucosal vaccine delivery [108]. Encapsulation of DOX in CNPs notably improved drug targeting and drug permeability, as demonstrated by Zare et al., showing a 12.7-fold increase in intestinal absorption. Paclitaxel-loaded CNPs demonstrated stronger anticancer effects than free paclitaxel in MDA-MB-231 breast cancer cells, with sustained release and improved cytotoxicity. Studies also show pH-responsive release and enhanced uptake of 5-FU-loaded CNPs in colorectal cancer models [109].

A study by Shali et al. [110] examined the co-delivery of insulin-like growth factor 1 receptor (IGF-1R)-specific small interfering RNA (siRNA) and DOX using chitosan-based NPs to improve anticancer efficacy in the A549 lung cancer cell line. The CNPs had an average size of approximately 176 nm along with a zeta potential of about 11 mV and a polydispersity index of 0.3, indicating their suitability for drug delivery. The results illustrated that the combination of IGF-1R siRNA and DOX encapsulated in CNPs possessed a synergistic effect, notably maximizing cytotoxicity and inducing apoptosis in A549 cells. The co-delivery system significantly downregulated cell migration and reduced the expression of vascular endothelial growth factor (VEGF), matrix metalloproteinase 9 (MMP9), and signal transducer and activator of transcription 3 (STAT3). All of these are associated with tumor progression and metastasis. These findings suggested that the chitosan-based NP system effectively increases the therapeutic efficacy of DOX by simultaneously silencing IGF-1R, a receptor implicated in cancer cell survival and proliferation. This dual-target approach holds beneficial results for improving lung cancer treatment outcomes by overcoming limitations associated with conventional chemotherapy.

Mahmood et al. [111] also demonstrated the synthesis and anticancer potential of selenium NPs (Se NPs) combined with CNPs (Se-CNPs) for lung cancer therapy. These NPs were synthesized by using grape seed extract, applying a green synthesis approach that is environmentally friendly and sustainable. Characterization of the NPs revealed spherical shapes with average sizes of 55.285 nm for SeNPs and 30.9 nm for Se-CNPs. Both types possessed dose-dependent cytotoxicity against the A549 lung cancer cell line, having IC50 values of 24.09 µg/mL for CNPs and 18.56 µg/mL for SeNPs. Also, normal human kidney (HK-2) cells were not significantly affected, which indicated the selective toxicity toward cancer cells. Mechanistic studies showed that treatment with SeNPs and Se-CNPs led to amplified generation of ROS, resulting in loss of membrane potential and mitochondrial dysfunction. These effects resulted in apoptosis of A549 cells via ROS-mediated pathways. The findings suggest that Se NPs and Se-CNPs can modulate ROS signaling, leading to cancer cell death. These results emphasize the potential of biogenic Se NPs and Se-CNPs as promising nanotherapeutic agents for lung cancer treatment, leveraging an eco-friendly alternative to conventional therapies.

Mahmoud and colleagues [112] investigated the development and properties of berberine-loaded chitosan nanoparticles (BBR-CNPs) and evaluated their efficacy in protecting male albino mice from urethane-induced lung cancer. The synthesized BBR-COSNPs exhibited a spherical morphology with an average size of 45.56 nm and a zeta potential of 39.82 mV, indicating excellent stability and suitability for drug delivery. In vivo studies demonstrated that BBR-COSNPs effectively inhibited lung tumor progression and promoted apoptosis by regulating serum BAX levels and caspase 9 gene expression in lung tissue. Additionally, these NPs reduced tumor angiogenesis by lowering serum VEGFR2 and suppressing HIF-1 gene expression in the lungs. The study suggests that BBR-COSNPs could be a promising oral anticancer therapy for lung cancer, offering an environmentally friendly alternative to traditional treatments. These findings underscore the potential of BBR-COSNPs as an effective nanotherapeutic approach for lung cancer management.

Zhu et al. [113] examined the targeting efficacy of T7 peptide-modified (CBT) NPs was examined through a fluorescence uptake study involving six experimental groups (non-modified CB NPs and targeted CBT NPs at 30 and 60 min, and pretreated CB/CBT with free T7 at 60 min). Flow cytometry amplified fluorescence uptake, thereby revealing that CBT at 60 min possessed the highest intensity. To confirm the specificity of T7-mediated targeting, cells were pretreated with excess free T7 peptide, that notably reduced CBT uptake. Immunofluorescence imaging of lung cancer cells (A549 and H1299) validated the findings. The fluorescence distribution pattern supported increased cellular uptake due to the T7 targeting peptide.

Patel et al. [114] conducted a study aimed at developing a targeted pulmonary drug delivery system for lung cancer treatment using silibinin-loaded CNPs functionalized with folic acid NPs. The researchers aimed to increase the therapeutic efficacy and site-specific delivery of silibinin by utilizing a quality by design approach, a natural anticancer agent, by creating inhalable NPs that actively target folate receptors, which are highly expressed in many lung cancer cells. Folic acid was electrostatically conjugated to CNPs to achieve targeted delivery. The optimized formulation demonstrated promising physicochemical characteristics, including a spherical morphology, an average particle size of 275 ± 1.20 nm, a polydispersity index of 0.234 ± 0.07, and a positive surface charge (ζ-potential) of 32.50 ± 0.21 mV, which is favorable for cellular uptake. The drug-loading and entrapment efficiency were satisfactory, with 75.52 ± 0.87% of silibinin encapsulated within the NPs. In vitro release studies showed a cumulative drug release of 63.25 ± 1.21% over 48 h, indicating sustained drug delivery. The aerodynamic behavior of the formulation was assessed to ensure its suitability for pulmonary administration with a mass median aerodynamic diameter of 2.75 ± 1.02 µm and a geometric size distribution of 3.15 ± 0.88 µm in the optimal range for deep lung deposition. The cytotoxicity assay conducted on a lung cancer cell-bearing rat model indicated an IC_50_ value of 24.5 µg/mL after incubating for 24 h, suggesting notable anticancer potential. The biodistribution studies confirmed that silibinin-loaded CNPs functionalized with folic acid NPs led to a higher accumulation of silibinin in lung tissues compared to non-targeted formulations. These results collectively show that the developed NPs system not only offers a non-invasive delivery method but also confirms improved localization of the therapeutic agent at the tumor site due to active targeting, making it a promising candidate for lung cancer therapy.

### 4.2. Sodium Alginate-Based Nanoparticles

Polymeric biomaterials like alginate (ALG) are widely used in medical applications due to their compatibility with physiological conditions and lesser adverse effects. ALG is highly valued for its biocompatible and hemostatic properties, as well as its capacity for chemical modification. Its hydroxyl and carboxyl groups allow hydrogen bonding, making it suitable for gel formation, muco-adhesion, and improving transdermal drug delivery [115]. ALG NPs are effective drug carriers, allowing biodegradability, high drug-loading potential, and low toxicity. These NPs can seal a variety of therapeutic agents, including oligosaccharides, proteins, and anticancer compounds. They are adaptable for multiple administration routes—oral, nasal, intravenous, and ocular [115]. ALG’s properties can be improved through blending with other polymers, chemical or physical crosslinking, or surface modifications using targeting agents such as antibodies, peptides, or aptamers. These modifications enhance specificity and minimize required drug doses [115].

Huang et al. [116] formulated an innovative nanocomposite comprising silver NPs (Ag NPs) supported on alginate-modified magnetic NPs (Fe_3_O_4_/Alg). The material was synthesized to target human lung carcinoma cells. The use of sodium alginate, which is a natural anionic polysaccharide, provided stability and biocompatibility during the reduction of Ag(I) ions to Ag NPs. The resulting nanocomposite combines the therapeutic potential of the magnetic targeting capability of Fe_3_O_4_ with silver. Given the high mortality rate of lung cancer and limitations in current treatments, this study shows a promising magnetically controllable, biocompatible, and cytotoxic agent, particularly designed to oppose lung cancer cells without harming normal cells. The synthesis of the Fe_3_O_4_/Alg-Ag nanocomposite began with the preparation of magnetite (Fe_3_O_4_) particles, served as the magnetic core. These particles were coated with sodium alginate, which functioned as both a stabilizer and reducing agent for silver ions. Then, after AgNO_3_ was introduced, AgNPs were reduced in situ, resulting in their stable deposition on the alginate-modified Fe_3_O_4_ surface. The resulting composite was characterized by various techniques; Fourier Transform Infrared (FTIR) confirmed chemical bonding and functional groups Field Emission Scanning Electron Microscopy (FE-SEM) and Transmission electron microscopy [117] offered morphological insights, Energy Dispersive X-ray (EDX) analysis validated elemental composition, Vibrating Sample Magnetometer (VSM) evaluated magnetic properties, and inductively coupled plasma–optical emission spectrometer (ICP-OES) provided quantitative elemental analysis. To evaluate biological effects, cytotoxicity studies were conducted using the 3-(4,5-dimethylthiazolyl-2)-2,5-diphenyltetrazolium bromide (MTT) assay. Non-small cell lung carcinoma cell lines NCI-H1975, NCI-H1563, and NCI-H1299 were treated with varying concentrations of the nanocomposite. The MTT assay assessed mitochondrial activity by measuring absorbance at 570 nm. Human umbilical vein endothelial cells (HUVECs) were used as a control to detect toxicity toward normal cells. The nanocomposite’s antioxidant activity was analyzed by using the 2,2-(2,2-diphenyl-1-picrylhydrazyl (DPPH) assay, in which half of the free radicals were neutralized at a concentration of 194 μg/mL, demonstrating moderate antioxidant capacity, potentially linked to its anticancer effects.

Recently, Işıklan et al. [118] formulated an innovative alginate-based bio-nanocomposite for targeted delivery of the chemotherapeutic drug etoposide and improved photothermal therapy in lung cancer treatment. The research highlights the central role of alginate in creating a stable, responsive delivery platform. Chemical modifications in alginate were performed by grafting it with poly (2-hydroxypropyl methacrylamide), forming a copolymer (SA-g-poly (2-hydroxypropyl methacrylamide) that improved the structural and functional features of the nanocomposite. The (SA-g-poly (2-hydroxypropyl methacrylamide) matrix was further combined with magnetite-functionalized graphene oxide to enhance responsiveness to external stimuli. However, it is the alginate backbone that provides the primary scaffold, ensuring the hydrogel maintains its integrity and allows for environmentally sensitive drug release. The nanocomposite was synthesized by using an emulsion method and underwent comprehensive physicochemical characterization using FT-IR, ultraviolet–visible (UV–Vis) spectroscopy, X-ray diffractometer (XRD), dynamic light scattering (DLS), TEM, FE-SEM, and atomic force microscope (AFM) analyses. These techniques confirmed the successful formation of a nanostructured network with favorable morphological and surface properties. Upon exposure to near-infrared laser light (808 nm, 1 W/cm^2^, 10 min), the composite possessed a notable temperature rise of over 29 °C. This thermal response facilitated a controlled release of etoposide, further regulated by pH, magnetic fields, and magnetite-functionalized graphene oxide concentration. The alginate-based platform played a vital role in enabling stimulus-responsive drug release, making it especially effective for site-specific lung cancer therapy. Cell studies using H1299 lung cancer lines revealed that the nanocomposite, when combined with near-infrared irradiation, notably increased cell death, demonstrating the synergistic potential of combined chemotherapy and photothermal treatment.

### 4.3. Gelatin-Based Nanoparticles

Gelatin-based NPs (GNPs) offer significant benefits in the management of lung cancer due to their biodegradability, biocompatibility, and ease of surface modification. Derived from collagen, gelatin is a naturally occurring polymer that is well tolerated and non-toxic in biological systems, making it an ideal material for drug delivery applications. GNPs can encapsulate a vast range of therapeutic agents, including targeted therapies, chemotherapeutics, and immunomodulators, allowing for controlled and sustained release at tumor sites [119]. In lung cancer, targeted delivery is especially critical to reduce damage to healthy lung tissue and minimize systemic side effects. One major benefit of GNPs is their ability to increase drug solubility and stability, thus improving the bioavailability of poorly soluble anticancer drugs. Their flexible surface properties allow for functionalization with antibodies and ligands, enabling active targeting of cancer cells or tumor-associated microenvironments [120]. This targeted approach enhances therapeutic efficacy and minimizes off-target toxicity. GNPs can be engineered to respond to specific stimuli such as pH or enzymes present in tumor tissues, allowing site-specific drug release. Their small size promotes penetration into tumors and increases cellular uptake. Overall, GNPs display a promising and versatile platform for safer, more effective lung cancer therapy [121].

Ali et al. [122] performed a novel approach to improve cancer immunotherapy by using GNPs as carriers for nivolumab, which is a PD-1 checkpoint inhibitor, to promote the cytotoxic activity of effector Jurkat T-cells against A549 lung carcinoma cells. The researchers developed GNPs to increase the delivery of nivolumab for improved cancer immunotherapy. The optimized GNPs (191.9 nm, PDI 0.027, 54.67% entrapment) showed increased cytotoxicity in a Jurkat T-cell–A549 lung carcinoma co-culture. Nivolumab-loaded GNPs achieved an 87.88% inhibition rate versus 60.53% for the free drug and a lower IC_50_ (0.41 µM vs. 1.22 µM), indicating increased potency. This nano-formulation allows a promising, more effective, and safer alternative for targeted cancer immunotherapy.

Gu et al. [94] formulated paclitaxel-loaded PEGylated GNPs (PEG-PTX-GNPs) to improve treatment efficacy in NSCLC. First, PTX-GNPs were synthesized using glutaraldehyde crosslinking and then PEGylated with PEG 400. These NPs were evaluated for surface morphology, size, drug loading, in vitro release, zeta potential, cytotoxicity, cellular uptake, in vivo antitumor activity, histopathology, pulmonary deposition, and immunohistochemistry. The PEG-PTX-GNPs were spherical (90–115 nm) with a drug loading of 20.18–32.11% and stable encapsulation efficiency. In vitro, they showed the highest antiproliferative effect and cellular uptake. In vivo, they achieved 100% survival, maximum tumor inhibition, highest pulmonary deposition (6.5–12.55 μg/g), and superior histological outcomes, confirming their therapeutic potential in NSCLC.

Vaghasiya et al. [123] formulated an enzyme-responsive, advanced, and receptor-targeted drug delivery system using GNPs for effective lung cancer therapy. These NPs were designed to respond particularly to the TME by targeting overexpressed mannose receptors and matrix metalloproteinase-2 (MMP-2) enzymes commonly found in NSCLC tissues. The GNPs were surface-functionalized with concanavalin A, a plant lectin that binds to mannose-rich glycoproteins on cancer cells or tumor cells membranes, increasing receptor-mediated endocytosis. Cisplatin was encapsulated within the gelatin matrix to form CG-NP, and when coated with concanavalin-A, the final construct was termed CCG-NP. This NP system possessed a dual-targeting mechanism as concanavalin-A allowed cell-specific uptake via mannose receptor recognition, and the gelatin matrix was engineered for MMP-2-sensitive degradation, leveraging enzyme-triggered and localized release of cisplatin. In vitro studies using A549 lung cancer cells showed that CCG-NPs possessed superior cellular internalization after 12 h compared to non-coated particles. The presence of MMP-2 boosted drug release, enhancing therapeutic outcomes. Also, CCG-NPs notably induced ROS production, induced apoptosis, and caused cell cycle arrest in S and G2/M phases. Overall, this stimuli-responsive, inhalable, NP system provides a highly selective and efficient platform for lung cancer treatment.

**Figure 3:**
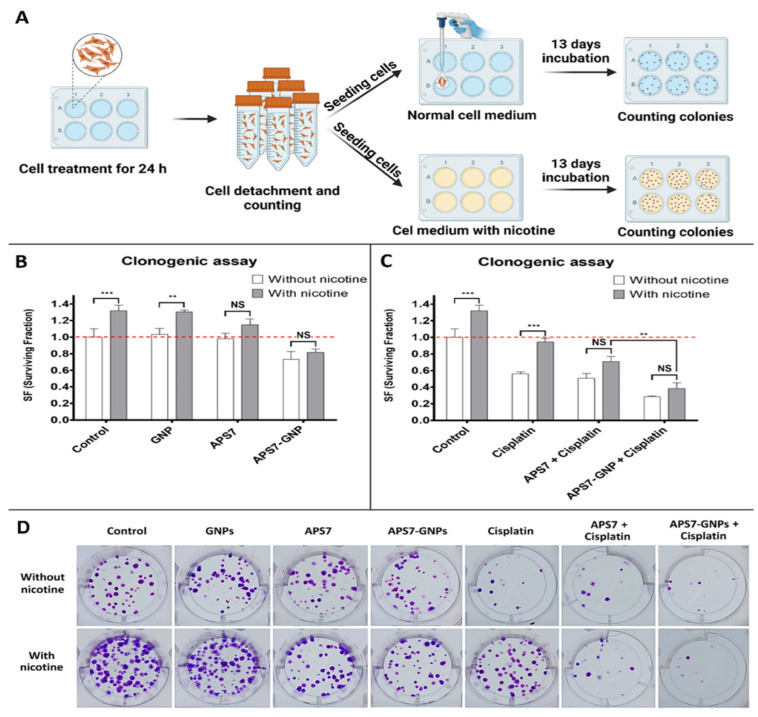
Effects of APS7-GNPs and cisplatin on colony formation of A549 lung cancer cells in normal and nicotine-containing conditions. (**A**) Schematic illustration of the clonogenic assay procedure. Cells were treated with gold nanoparticles (GNPs), APS7, APS7-GNPs, or cisplatin for 24 h, with or without nicotine, followed by seeding and incubation for 13 days before colony counting. (**B**) Quantitative analysis showing that APS7-GNPs significantly reduced colony formation compared to GNPs or APS7 alone, especially under nicotine exposure. (**C**) Combination of APS7-GNPs with cisplatin further reduced surviving fraction (SF), even in nicotine-containing medium. (**D**) Representative images of colony formation under each treatment condition. ** equals *p* < 0.01; *** equals *p* < 0.001; NS equals *p* > 0.05—not significant; ANOVA with Bonferroni’s post test). Data from [124], published by Elsevier, 2024.

Kononenko et al. [124] examined a novel lung cancer treatment strategy using GNPs as carriers for APS7, which is a synthetic analog of a marine toxin that functions as a nicotinic acetylcholine receptor (nAChR) antagonist. Their study aimed to improve chemotherapy efficacy while reducing off-target effects, mainly in nicotine-exposed lung cancer cells, which often show high resistance to therapy due to nAChR activation. Nicotine, by stimulating these receptors, supports cancer cell proliferation and inhibits apoptosis. By administering APS7 through GNPs, the researchers targeted these resistance mechanisms in A549 lung adenocarcinoma cells, displaying a potential dual-action therapeutic nanoplatform. APS7 was encapsulated within GNPs (APS7-GNPs) and their biological effects were tested on A549 lung cancer cells, including in nicotine-stimulated conditions. The study assessed calcium influx, proliferation, and chemosensitivity to cisplatin. Both APS7 and APS7-GNPs blocked nicotine-induced calcium influx and improved cisplatin efficacy. APS7-GNPs notably outperformed free APS7 by more effectively reducing proliferation and demonstrating higher selectivity, sparing normal lung epithelial BEAS-2B cells. The primary method to measure reproductive survival and colony formation was a clonogenic assay, assessing the influence of nicotine, APS7, and cisplatin on cancer cell viability over 13 days. The panel in Figure 3A evaluated the impact of nicotine on untreated and pretreated A549 cells utilizing a clonogenic assay. Cells exposed to 0.5 µM nicotine displayed notably higher surviving fractions (SFs) as compared to those grown in nicotine-free medium, indicating nicotine’s pro-survival effect. This suggests that nicotine improves the reproductive viability of lung cancer cells, which may contribute to chemoresistance. Figure 3B shows the effects of pre-treatment with GNPs, APS7, and APS7-GNPs on reproductive survival were examined. Pre-treatment with empty GNPs had no significant effect on SF in either medium. Free APS7 reduced the SF of nicotine-stimulated cells but not in standard medium. Significantly, APS7-GNPs notably minimized SF in both media, suggesting increased efficacy and a more generalized antiproliferative effect. Figure 3C displays a panel that assessed cisplatin’s impact, alone and in combination with APS7 or APS7-GNPs. Cisplatin alone reduced SF in normal conditions (SF = 0.56), but its effect was negligible under nicotine exposure (SF = 0.94). When combined with either APS7 or APS7-GNPs, this nicotine-induced resistance was attenuated. APS7-GNPs were more effective, restoring cisplatin sensitivity even in the presence of nicotine. Figure 3D presents imaging of colonies, further validating APS7-GNPs’ superior performance.

In 2020, Chen et al. [125] performed a study to address the challenge of cisplatin resistance in lung cancer therapy by developing a dual-drug delivery system using gelatin-based NPs. Cisplatin is a standard chemotherapeutic agent in treating lung cancer, but its efficacy is hindered by the development of resistance. To counter the limitation, the researchers designed a nanocarrier system incorporating both cisplatin and epigallocatechin gallate, a naturally occurring polyphenol known for its anticancer properties. Gelatin was selected as the polymeric matrix due to its inherent biocompatibility, biodegradability, and capacity for drug encapsulation, making it an ideal vehicle for targeted drug delivery. The self-assembled gelatin/epigallocatechin gallate (GE) NPs were synthesized first, followed by the incorporation of cisplatin to create GE NPs. These particles were spherical with an average size of 75 nm and a positive zeta potential of +19.83 ± 0.25 mV, as determined by dynamic light scattering and confirmed through TEM. The NPs showed high drug-loading efficiency, approximately 63.7% for cisplatin and 89% for epigallocatechin gallate. In vitro experiments using A549 human lung adenocarcinoma cells showed that GE-Pt NPs at low concentrations, i.e., epigallocatechin gallate 5 μg/mL and cisplatin 2 μg/mL, possessed notably improved cytotoxicity as compared to cisplatin alone. Further analysis via inductively coupled plasma mass spectrometry displayed higher intracellular accumulation of cisplatin when delivered through the NPs, attributed to efficient endocytic uptake, and this improved delivery led to increased therapeutic efficacy. The combination of epigallocatechin gallate and cisplatin within a gelatin-based nanocarrier possessed a synergistic effect, suggesting a promising approach to overcome cisplatin resistance. The study emphasizes the advantages of gelatin as a nanocarrier, not only for its safe biological profile but also for its ability to improve drug stability and promote controlled release. Overall, the research supports the potential of GE-Pt NPs as an effective strategy for enhancing lung cancer treatment outcomes.

### 4.4. Poly Lactic Acid-Based Nanoparticles

Poly lactic acid (PLA)-based NPs are biocompatible and biodegradable carriers derived from renewable sources, mainly used in drug delivery applications, including cancer therapy. In the management of lung cancer, PLA NPs offer a major platform for the targeted delivery of gene therapies, chemotherapeutic drugs, and immune-modulating agents. Their biodegradability ensures safe breakdown into lactic acid, a naturally occurring metabolite, minimizing long-term toxicity [126]. The primary advantage of PLA-based NPs lies in their ability to provide controlled and sustained drug release, which increases therapeutic efficiency while minimizing dosing frequency. Their small size facilitates deep tumor penetration and cellular uptake, while surface modification allows active targeting of lung cancer cells or tumor-specific receptors. PLA NPs can also protect sensitive drugs from premature degradation, improving their bioavailability [126]. Overall, PLA-based NPs systems increase treatment precision, reduce systemic side effects, and support the development of safer and more effective therapies for lung cancer patients.

**Figure 4:**
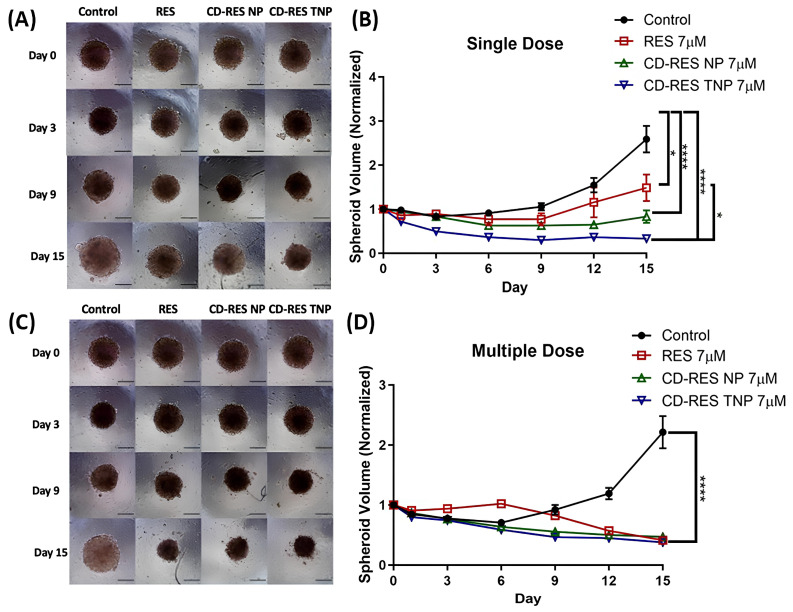
Three-dimensional spheroid assay evaluating the impact of treatments on H1299 tumor spheroid growth. (**A**) Representative images from a single dose. (**B**) Quantitative analysis of spheroid volume, normalized to day 0, for the single-dose group over 15 days. (**C**) Multiple-dose experiments captured at 10× magnification on days 0, 3, 9, and 15 (scale bar = 500 μm). (**D**) Volume quantification for multiple-dose spheroids during the same period. Significance between groups was analyzed by one-way ANOVA and Tukey’s multiple comparisons test. * *p* < 0.05 and **** *p* < 0.0001. Reproduced with permission from Xuechun Wang et al., Journal of Drug Delivery Science and Technology; published by Elsevier, 2025 [127].

Wang et al. [127] introduced a novel targeted drug delivery system using transferrin-conjugated polymeric NPs (CD-RES TNPs) to increase resveratrol (RES) delivery for the treatment of NSCLC. RES is a natural polyphenol with anticancer properties, but it has poor solubility and bioavailability. By complexing RES with cyclodextrin (CD) and encapsulating it in poly lactic glycolic acid (PLGA) NPs, functionalized with transferrin ligands, the researchers aimed to improve RES targeting to NSCLC cells via transferrin receptor (TfR)-mediated endocytosis, thereby enhancing therapeutic outcomes and reducing off-target toxicity. The researchers formulated cyclodextrin-RES-loaded NPs (CD-RES NPs) and CD-RES TNPs using PLGA and tested them in H1299 NSCLC cells. NPs demonstrated high encapsulation efficiency ~90%. Cytotoxicity assays indicated that CD-RES TNPs notably reduced IC_50_ values and colony growth compared to plain RES or non-targeted NPs. Apoptosis induction and anti-migration effects were improved in CD-RES TNPs. Hemolysis assays confirmed biosafety. Three-dimensional spheroid culture studies mimicking tumor architecture validated the superior efficacy of CD-RES TNPs in tumor penetration and growth inhibition over 15 days, under both single and multiple dosing regimens. Figure 4A shows single-dose spheroid images: After one dose of 7 μM treatment on day 0, spheroids were imaged over 15 days. Control spheroids showed continuous growth, while CD-RES NP and CD-RES TNP treatments significantly inhibited spheroid expansion. CD-RES TNPs notably reduced spheroid size more than plain RES, indicating enhanced tumor inhibition from receptor-mediated delivery. Figure 4B shows single-dose spheroid volume data: Quantitative data showed control spheroids grew 2.59 ± 0.30-fold (≈258.7%), while RES-treated spheroids grew 1.48 ± 0.30-fold (≈148.4%). CD-RES NP and CD-RES TNP-treated spheroids shrank notably, with volumes decreasing to 15.6 ± 14.0% and 67.1 ± 3.1% of the original size, respectively. CD-RES TNPs outperformed plain RES significantly (*p* < 0.05), though no statistical difference was found between CD-RES NP and CD-RES TNP groups. Figure 4C displays multiple-dose spheroid images: In a repeated-dose setup, spheroids treated with all formulations (RES, CD-RES NP, CD-RES TNP) possessed visibly reduced growth compared to controls. Visual observation confirmed a consistent pattern of volume suppression by NP treatments over 15 days. Figure 4D displays multiple-dose spheroid volume data: Control spheroids expanded 2.21 ± 0.27-fold (~221.5%), while RES, CD-RES NP, and CD-RES TNP groups reduced volumes to 58.3 ± 3.9%, 52.3 ± 4.0%, and 61.9 ± 3.6%, respectively (*p* < 0.0001 vs. control). This confirms the superior therapeutic efficiency of NP-based formulations in sustained tumor growth inhibition.

Yao et al. [128] conducted a study to improve lung cancer treatment by developing a novel nano-delivery system using PLGA NPs to co-deliver resveratrol and phosphatase and tensin homolog deleted on chromosome ten (PTEN) siRNA. NSCLC contributes to a majority of lung cancer cases are mainly metastatic, and survival rates remain low because of limited therapeutic efficacy and cancer cell resistance. Resveratrol, a naturally occurring polyphenol, possesses significant anticancer properties, including prevention of carcinogenesis, inhibition of tumor proliferation, and induction of apoptosis. However, its clinical application is restricted because of poor bioavailability and short biological half-life. To overcome this, the researchers encapsulated resveratrol along with PTEN-targeting siRNA into PLGA NPs modified with polyethylene imine (PEI). PLGA was selected due to its favorable characteristics—biocompatibility, biodegradability, excellent drug-loading capacity, and extended systemic circulation, making it an ideal vehicle for controlled and passive tumor-targeted drug delivery. The NPs were synthesized using a solvent-free evaporation technique without stabilizers and were then characterized using electron microscopy. Resveratrol and siRNA were fluorescently labeled with Oregon Green and Cy5, respectively, to help in tracking and cellular uptake analysis. The final NPs complexes, termed PLGA-PEI-RES-PTEN, measured approximately 80 nm in size. Notably, NPs’ size increased upon PEI surface modification and siRNA loading. Functional studies, including Western blotting and CCK8 assays, indicated that co-delivery of resveratrol and PTEN siRNA enhanced cytotoxicity against A549 and cisplatin-resistant A549/T12 lung cancer cell lines. The knockdown of PTEN notably amplified resveratrol’s toxicity toward cancer cells, suggesting a synergistic anticancer effect. Additionally, the nanocomplex was effectively internalized by cancer cells, facilitating PTEN gene silencing and thereby disrupting tumor cell survival and proliferation. The study demonstrated that this PLGA-based nanoplatform not only improved the therapeutic efficacy of resveratrol but also provided a dual-action approach by combining chemotherapeutic and gene silencing effects.

Chittasupho and colleagues demonstrated that conjugating the CXCR4-targeting peptide LFC131 onto PLGA NPs significantly enhanced the delivery of doxorubicin to CXCR4-expressing A549 lung cancer cells. These LFC131-DOX NPs exhibited faster and greater cellular uptake, CXCR4-specific internalization confirmed via competitive inhibition, and sustained drug release, highlighting their promise as a targeted and controlled delivery system [83]. In another study, the authors demonstrated that cLABL-conjugated PLGA NPs specifically target ICAM-1-expressing A549 lung epithelial cells, enhancing doxorubicin uptake and cytotoxicity via ICAM-1-mediated internalization. The system showed sustained drug release and confirmed potential as a targeted and controlled-release drug delivery platform [84].

### 4.5. Poly Caprolactone-Based Nanoparticles

Polycaprolactone (PCL)-based NPs are a class of biodegradable and biocompatible polymeric carriers known for their slow degradation rate and excellent drug encapsulation capabilities. In the context of lung cancer management, PCL NPs have emerged as a valuable platform for the sustained and targeted delivery of anticancer agents. Their hydrophobic nature allows effective loading of poorly water-soluble chemotherapeutic drugs, enhancing their solubility and therapeutic impact [129]. The key advantage of PCL NPs is their long-term drug release profile, which maintains therapeutic drug levels over extended periods, reducing the need for frequent dosing. This sustained delivery helps minimize systemic toxicity and enhances patient compliance. Additionally, PCL NPs can be surface-functionalized for active targeting of lung tumor cells, improving drug accumulation at the tumor site while sparing healthy tissue. Their stability and compatibility with a range of drugs make PCL NPs a promising tool for improving treatment outcomes in lung cancer therapy [130].

**Figure 5:**
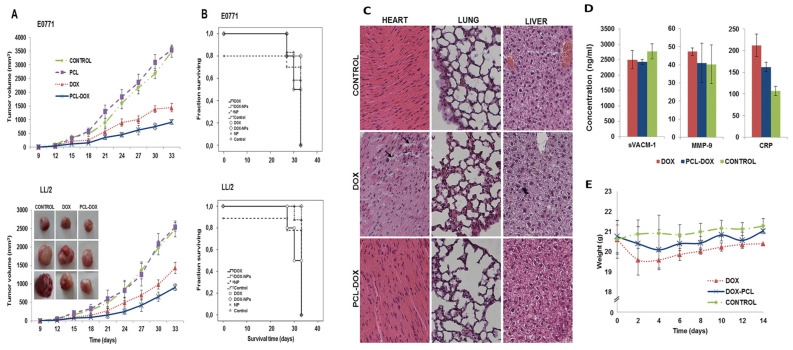
(**A**) Tumor volume progression in C57BL/6 mice with subcutaneous tumors derived from E0771 breast cancer and LL/2 lung cancer cells is shown after intravenous treatment with DOX-loaded PCL NPs, free DOX, and blank PCL NPs. Tumor growth inhibition is graphically presented as mean ± S.D. (*n* = 10), with representative images of LL/2 tumors. (**B**) Kaplan–Meier survival curves depict the survival outcomes of mice following the same treatments. Statistical differences between groups were analyzed using the log-rank test (*n* = 10). (**C**) Hematoxylin and eosin staining of heart, liver, and lung tissues reveals cardiotoxic effects in mice treated with free DOX. (**D**) Blood biomarkers of cardiovascular injury show elevated CRP levels in the free DOX group. (**E**) Body weight trends over 14 days post-treatment show weight loss in mice given free DOX. Reproduced with permission from Laura Cabeza et al., European Journal of Pharmaceutical Sciences; published by Elsevier, 2017 [131].

Cabeza et al. [131] developed DOX-loaded PCL NPs as a strategy to enhance antitumor efficacy while reducing systemic toxicity in lung and breast cancer treatments. Recognizing the severe side effects of free DOX, especially cardiotoxicity, the researchers designed PCL NPs with improved physicochemical and biocompatibility profiles. Their goal was to assess these NPs’ ability to deliver DOX more effectively to tumor sites while sparing healthy tissues, using both in vitro and in vivo models, including human and mouse breast and lung cancer cell lines, as well as immunocompetent mice bearing tumor xenografts. In their experimental setup, the team synthesized DOX-loaded PCL NPs via a modified nanoprecipitation method and characterized them for cellular uptake, cytotoxicity, and in vivo therapeutic effect. The NPs displayed excellent biocompatibility, enhanced DOX uptake by cancer cells, and induced up to a 98% reduction in the IC_50_ in E0771 breast cancer cells. In vivo studies confirmed a significant reduction in tumor volume (~36%) in both breast and lung tumor models. Importantly, mice treated with DOX-PCL NPs maintained normal body weight and showed fewer toxic side effects, particularly reduced cardiotoxicity, compared to free DOX-treated groups. Figure 5A demonstrates tumor volume in C57BL/6 mice. No significant difference was seen between saline and blank PCL NP groups. However, mice treated with DOX-PCL NPs showed a ~36% greater tumor volume reduction than those treated with free DOX, in both lung and breast cancer models (*p* < 0.05), indicating superior antitumor efficacy of the nano-formulation. Figure 5B highlights survival data. In lung tumor-bearing mice, DOX-PCL NP treatment significantly prolonged survival compared to free DOX. However, no survival difference was observed between the DOX and DOX-PCL NP groups in breast cancer models, as shown by overlapping Kaplan–Meier curves. Figure 5C shows organ-specific toxicity. Histological examination of liver and lung tissues revealed no notable injuries across all treatment groups, confirming the safety of PCL NPs at the administered dose. Figure 5D focuses on cardiotoxicity markers. Free DOX elevated CRP levels by 49.7% (*p* < 0.05), indicating inflammation, while DOX-PCL NPs caused no such increase. Other markers like MMP-9 and sVACM-1 remained stable across all groups. Figure 5E depicts body weight trends during a 14-day study. No significant weight changes were observed in any group, further supporting the minimal systemic toxicity of DOX-loaded PCL NPs.%

Akbari et al. [132] analyzed a novel dual-drug nanocarrier system to improve the therapeutic efficacy of lung cancer treatment by co-encapsulating cisplatin and methotrexate (MTX) within a PLA-PEG copolymer-based NPs platform. The study aimed to harness the complementary mechanisms of these two clinically established anticancer agents. Cisplatin induces DNA damage leading to apoptosis, while MTX disrupts folate metabolism essential for DNA synthesis and repair. The combination of these drugs within a single delivery system offers a strategic advantage in targeting multiple pathways involved in cancer progression. The selected PLA-PEG polymeric matrix was chosen due to its biodegradable, biocompatible, and environment-responsive properties, making it an ideal candidate for controlled and sustained drug release in cancer therapy. The NPs, characterized as spherical via scanning electron microscopy, effectively encapsulated both drugs. In vitro evaluation against A549 human lung carcinoma cells demonstrated that the methotrexate-*co*-encapsulating cisplatin-loaded NPs notably reduced cell viability, as confirmed by MTT assay, and induced significant levels of apoptosis, as observed through DAPI nuclear staining. Further, qRT-PCR analysis supported these findings by revealing enhanced expression of apoptosis-related genes. This dual-drug-loaded nanocarrier exhibited superior antitumor activity compared to single-drug formulations, highlighting its potential in overcoming chemoresistance and enhancing cytotoxic effects. The synergistic action of MTX and co-encapsulating cisplatin, delivered through a PLA-PEG platform, not only maximizes therapeutic impact but also reduces systemic toxicity by allowing for targeted and localized drug release. The study underlines the critical role of PLA-PEG copolymers in nanomedicine, as they provide a stable, customizable, and efficient vehicle for delivering combination chemotherapy agents. These findings suggest that PLA-PEG-based NPs co-loaded with methotrexate and cisplatin represent a promising and effective approach for the treatment of NSCLC, warranting further preclinical and clinical exploration.

### 4.6. Poly(amidoamine)-Based Nanoparticles

Poly(amidoamine) (PAA or PAMAM) could be stepwise synthesized to yield dendrimers that have a hyper-branched, tree-like structure with active functional groups and well-defined size. Dendrimers are biocompatible, water soluble, and redox sensitive. Regrettably, uncoated dendrimers were reported to have certain cellular toxicity; therefore, surface modification, such as PEGylation, is essential to improve their safety [133].

Guo et al. [134] synthesized PAA-iRGD/siEGFR polyplexes from PAA dendrimers that were functionalized with iRGD tumor penetration peptide and complexed with siEGFR, siRNA that silences EGFR for lung cancer therapy. PAA-iRGD showed an average size of 223.7 ± 3.1 nm and zeta potential of 35.2 ± 3.2 mV. PAA-iRGD/siEGFR showed higher gene silencing ability compared to PAA and PEI, significantly inhibited H1299 cells’ proliferation and migration. PAA-iRGD/siEGFR inhibited lung tumor growth in BALB/c mice compared to PAA/siEGFR and PEI/siEGFR. These findings indicated that PAA-iRGD is a promising gene carrier in cancer treatment.

Bai et al. [135] prepared docetaxel (DTX)-alendronate (ALN)-PAMAM (DTX@ALN-PAMAM) to treat bone metastases of lung cancer. The drug loading, size, and zeta potential of DTX@ALN-PAMAM were 5.2 ± 0.4%, 99 ± 13 nm, and 4.35 ± 0.28 nm, respectively. DTX@ALN-PAMAM significantly enhanced DTX anticancer activity and inhibited osteoclast formation in in vitro bone metastases model. DTX@ALN-PAMAM showed the highest cancer inhibition rate compared to ALN and free DTX in vivo mouse model. The findings suggested that DTX@ALN-PAMAM exhibited synergistic effects that could be used on bone metastases of lung cancer.

### 4.7. Polymethacrylate-Based Nanoparticles

NPs from poly (methyl methacrylate) (PMMA) are easy to synthesize and functionalize, biocompatible. Core–shell PMMA NPs were developed to resolve the agglomeration issue of PMMA. Yin et al. [136] prepared polymethacrylic acid (PMAA) NPs loaded with Fe(III) and cypate dye coated with mesenchymal stem cell membrane (MSCs) or red blood cell membranes (RBCs) for photothermal-enhanced radiotherapy of NSCLC. The average size and zeta potential of Cyp-PMAA-Fe@MSCs were 248.4 and −22.3 mV. Cyp-PMAA-Fe@MSCs exhibited a strong photothermal hyperthermia effect when exposed to 808 nm laser irradiation. The fluorescence signal of Cyp-PMAA-Fe@MSCs in tumor-bearing mice was 21% stronger than that in the RBCs group, indicating more tumor accumulation. Tumors on mice treated with photothermal therapy and radiotherapy shrank 32% more than those treated with only radiotherapy. The findings suggested that Cyp-PMAA-Fe@MSCs could be applied for tumor diagnosis and precise treatment of NSCLC. Table 5 illustrates the recent investigation of polymeric NPs for the treatment of lung cancer.

## 5. Clinical Trials Associated with Polymeric NPs-Based Drug Delivery for Lung Cancer

Clinical trials investigating polymeric NPs for lung cancer treatment are gaining momentum due to their potential to enhance targeted drug delivery and reduce systemic toxicity. Several ongoing and completed trials have evaluated polymeric NP-based formulations such as albumin-bound paclitaxel (Abraxane) and PEGylated drug carriers for improved therapeutic outcomes [138]. These trials focus on evaluating safety, pharmacokinetics, and tumor response in patients with NSCLC. Early results indicate improved drug accumulation at tumor sites and reduced adverse effects compared to conventional therapies. However, larger-scale, randomized studies are needed to establish their efficacy and enable widespread clinical adoption. Table 6 illustrates the summary of clinical trials on polymeric NPs for lung cancer management.

## 6. Challenges, Limitations, and Future Perspective

Despite the promising advancements in polymeric NPs for targeted lung cancer therapy, several critical challenges and limitations hinder their full clinical translation. These challenges are associated with physicochemical properties, biological barriers, safety concerns, manufacturing scalability, and regulatory approval. The major obstacle for polymeric NPs mediated lung cancer therapy is the complex biological environment that impedes efficient drug delivery [143]. After systemic administration, NPs must navigate through the bloodstream, avoid clearance by the mononuclear phagocyte system, and overcome endothelial barriers to reach the tumor site. Even when utilizing passive targeting via the EPR effect, many NPs are sequestered by the liver and spleen, reducing lung-specific accumulation. Active targeting using ligands such as antibodies or peptides improves specificity, but receptor heterogeneity in tumors can limit their efficacy [144].

The EPR effect, while foundational to passive tumor targeting, is highly heterogeneous and influenced by multiple factors. Tumor type significantly affects vascular permeability, with highly angiogenic and leaky vasculature in some solid tumors (e.g., hepatocellular carcinoma) favoring stronger EPR, whereas desmoplastic or poorly vascularized tumors (e.g., pancreatic cancer) exhibit limited NP accumulation. Tumor size also plays a role—smaller, rapidly growing tumors often have immature vasculature that enhances permeability, while larger tumors may develop necrotic or hypoxic cores with reduced perfusion, limiting drug delivery [145]. Additionally, tumor location impacts EPR efficacy; for example, tumors in highly vascularized organs (like the liver) may exhibit different NP uptake patterns than those in low-perfusion sites or protected areas (such as the brain, due to the blood–brain barrier). These variabilities present challenges in clinical translation, as preclinical models often overestimate EPR-driven delivery efficiency, compared to human tumors, which are more heterogeneous. Understanding and accounting for these factors is essential for designing nanomedicines with consistent and predictable therapeutic outcomes [146] (Table 7).

While pulmonary (inhalation) delivery is ideal for localized lung cancer treatment, achieving uniform drug deposition in deep lung tissues remains difficult. Factors such as mucociliary clearance, enzymatic degradation, and variable breathing patterns impact drug retention and efficacy. Moreover, the formulation must maintain aerodynamic properties compatible with inhalation devices without compromising NP stability or drug activity [144].

Maintaining the physical and chemical stability of polymeric NPs during formulation, storage, and administration is another challenge. Premature drug leakage, aggregation, and degradation can reduce therapeutic efficiency. Ensuring controlled, stimulus-responsive drug release triggered by pH, enzymes, or redox conditions in the TME requires precise engineering, which is often difficult to reproduce consistently across batches [150].

Although polymeric materials like PLGA, chitosan, and PEG are generally biocompatible, the degradation products of some polymers may cause inflammation or toxicity. Furthermore, limited data exist on the long-term fate and clearance of NPs, especially when repeatedly administered. Accumulation in non-target organs and the risk of immunogenicity remain unresolved concerns. Surface modifications intended to prolong circulation or enhance targeting can inadvertently increase toxicity or alter pharmacokinetics unpredictably [151].

Translating lab-scale synthesis to large-scale production poses major technical and economic challenges. Reproducibility in NPs size, drug-loading efficiency, surface charge, and batch consistency is essential for clinical success. Sophisticated techniques such as microfluidics or nanoprecipitation, though precise, are often expensive or not easily scalable, limiting their commercial viability [152]. Moreover, from a health economics standpoint, cost-effectiveness is influenced by quality-adjusted life years gained. Preliminary pharmacoeconomic modeling in oncology suggests that targeted nanomedicine, despite higher upfront costs, can achieve favorable cost per quality-adjusted life years ratios when accounting for extended survival and improved quality of life. Additionally, the potential for multifunctional theranostic NPs combining therapy and real-time imaging could streamline diagnostic workflows, further reducing cumulative costs [153]. Nonetheless, manufacturing scalability, supply chain logistics, and regulatory compliance remain cost drivers. Without economies of scale and optimized production, these therapies risk being unaffordable in low- and middle-income countries. Health systems may require value-based pricing models and risk-sharing agreements to ensure accessibility [154]. In conclusion, while novel NP-based lung cancer therapies currently carry higher sticker prices than conventional modalities, their potential to deliver durable clinical benefits with fewer downstream costs positions them as economically viable in the long term provided manufacturing and policy innovations support widespread adoption [155].

The regulatory pathway for NPs-based drug delivery systems is still evolving. These systems often blur the lines between drugs and devices, complicating approval processes. Agencies such as the FDA and EMA require comprehensive characterization of physicochemical properties, stability, pharmacokinetics, biodistribution, and long-term safety. Demonstrating consistent manufacturing under Good Manufacturing Practice conditions is essential [156]. Additionally, lack of standardized evaluation protocols for NPs complicates the approval process. Regulatory submissions must address potential immunogenicity, accumulation in non-target tissues, and variability in patient responses due to tumor heterogeneity. Bridging preclinical promise to clinical reality will require harmonized guidelines, robust scalability strategies, and cost-optimized manufacturing pipelines [97]. Additionally, patient variability, tumor heterogeneity, and limited predictive animal models further complicate clinical trials [99].

The patient variability and tumor heterogeneity in lung cancer stem from diverse genetic mutations (e.g., EGFR, KRAS, ALK) and phenotypic traits, as well as differences in tumor microenvironments. These variations can significantly impact therapeutic response, making one-size-fits-all NP designs less effective. To address this, PNPs with active targeting ligands can be engineered for patient-specific profiles [151]. Molecular profiling through next-generation sequencing can guide the selection of ligands—such as antibodies, peptides, or aptamers—that bind receptors overexpressed in an individual’s tumor subtype. Multifunctional PNPs can also incorporate multiple ligands to simultaneously target heterogeneous cell populations within the same tumor, reducing the risk of resistant subclones. Additionally, stimuli-responsive PNPs can be tuned to exploit unique tumor microenvironment features (pH, enzymes, redox gradients) that differ between patients [157]. Modular PNP platforms further allow rapid swapping of targeting moieties and payloads, enabling personalized therapy adaptation during treatment. This precision design approach improves drug accumulation in genetically diverse tumors, enhances therapeutic efficacy, and mitigates off-target toxicity, ultimately bridging the gap between nanomedicine innovation and real-world patient variability [158].

The integration of polymeric NPs into lung cancer therapy holds immense promise for transforming current treatment paradigms. Future research must prioritize the formulation of multifunctional, stimuli-responsive NPs effective in co-delivering chemotherapeutics, gene therapies, and immunomodulators in a single platform [145]. Innovations in ligand engineering, particularly tumor-specific peptides and antibodies, will enhance active targeting, overcome tumor heterogeneity, and improve drug accumulation at tumor sites. Additionally, the design of inhalable NP formulations offers a non-invasive, localized approach to maximize therapeutic outcomes while minimizing systemic toxicity, a critical advancement for lung cancer care [159].

Emerging trends in bioinspired and green-synthesized NPs, including hybrid polymer-metallic systems, also warrant extensive exploration, given their potential for synergistic imaging and therapeutic applications [160]. Long-term safety evaluations, pharmacokinetics, and scalable manufacturing techniques must be refined to bridge the gap between laboratory innovation and clinical translation. Moreover, integrating artificial intelligence and machine learning for predictive modeling of NP behavior and personalized therapy optimization could redefine precision oncology in lung cancer. Ultimately, multidisciplinary collaboration among materials scientists, oncologists, and regulatory agencies will be essential to realize the full clinical potential of polymeric nanomedicine for effective, patient-tailored lung cancer management [161].

## 7. Conclusions

Lung cancer remains a leading therapeutic challenge due to its aggressive nature, late diagnosis, and limitations of conventional treatments. Polymeric NPs have turned up as promising tools for targeted drug delivery, offering ameliorated efficacy, tumor specificity, and reduced systemic toxicity. This review explores polymeric NPs, including chitosan, gelatin, alginate, poly lactic acid, and polycaprolactone, which enhance the delivery of chemotherapeutics, gene therapies, and immunomodulators. These nanocarriers protect drugs from degradation and improve bioavailability. Advanced designs, including ligand-conjugated, stimuli-responsive, and multifunctional NPs, have further improved therapeutic outcomes. Incorporating green-synthesized metallic NPs and quantum dots into polymeric platforms has introduced novel theranostics opportunities, combining imaging and treatment. However, barriers including NPs stability, biological interactions, long-term toxicity, and production scalability remain key challenges. Future directions include optimizing material properties, enhancing biocompatibility, and exploring inhalable formulations. Artificial intelligence-based therapy design may also accelerate personalized treatment development. In conclusion, polymeric NPs represent a transformative approach in lung cancer therapy. With continued interdisciplinary research and clinical validation, these systems could significantly improve treatment outcomes, survival rates, and quality of life for lung cancer patients globally.

## Figures and Tables

**Table 1:** Comparing conventional and novel therapies for lung cancer management.

Therapy Aspect	Conventional Therapies	Novel Therapies	References
Primary treatment types	- Surgery (lobectomy, pneumonectomy) - Chemotherapy (platinum-based) - Radiation therapy (external beam)	- Targeted therapy (EGFR inhibitor/PI3K inhibitor) - Immunotherapy (PD-1/PD-L1 inhibitors) - Stereotactic body radiotherapy (SBRT)	[40,43,44]
Mechanism of action	- Cytotoxic drugs kill rapidly dividing cells - Radiation induces DNA damage	- Targeted drugs block specific oncogenic pathways - Immunotherapy enhances T-cell antitumor activity	[13]
Response rates	- 20–30% for chemotherapy - 30–50% for radiation in early stages	- 60–80% for targeted therapy in mutation-positive cases - 20–40% durable responses with immunotherapy	[14]
Adverse reactions	- Bone marrow suppression - Nausea/vomiting - Radiation pneumonitis	- Immune-related adverse events (colitis, pneumonitis) - Skin rash with EGFR inhibitors	[45,46,47,48]
Personalization level	- Largely based on cancer stage and histology	- Requires molecular profiling (NGS) - Biomarker-driven (PD-L1, TMB)	[49,50,51,52]
5-Year survival benefit	- 15–20% for stage III chemo-radiation - 40–50% for early-stage surgery	- 25–30% for stage IV with targeted therapy - 15–25% long-term survivors with immunotherapy	[52,53,54,55]

**Table 2:** Comprehensive tabular summary of polymeric reduced green metallic nanoparticles (PRGM NPs) in lung cancer management.

Aspect	Key Findings	References
Targeted imaging	Folic acid-conjugated chitosan nanoparticles (fCNA) significantly enhanced protoporphyrin IX (PpIX) accumulation in colorectal cancer cells (HT29, Caco-2) via folate receptor-mediated endocytosis, improving tumor detection.	[72]
Chemotherapy	Mitochondria-targeted green AuNPs increased reactive oxygen species (ROS) 4.2-fold in H460 cells and reduced tumor volume by 72% in xenografts (5 mg/kg dose).	[73]
siRNA delivery	Chitosan-coated AuNPs delivered EGFR siRNA to A549 xenografts, reducing tumor growth by 65% (0.5 mg/kg, q3d × 4 weeks).	[74]
Combo therapy	Fol-LSMO NPs enable targeted hyperthermia with DOX release in breast cancer. Triggers apoptosis and autophagy via caspase/LC3-II pathways. Folate conjugation enhances uptake, reduces off-target effects.	[75]

**Table 3:** Comparative overview of key polymers, targeting approaches, and delivery routes for polymeric NPs in lung cancer therapy.

Category	Examples	Advantages	Limitations	References
Polymers	PLGA	Biodegradable and biocompatible; FDA-approved; tunable degradation; suitable for controlled and sustained release; compatible with multiple drugs	Acidic degradation products may cause local irritation; relatively slow drug release for hydrophilic drugs	[90]
	PEG	Improves hydrophilicity; prolongs circulation time by reducing opsonization; reduces immunogenicity; enhances stability	Can cause “accelerated blood clearance” on repeated dosing; non-biodegradable (requires clearance); potential hypersensitivity reactions	[91]
	Chitosan	Natural, biocompatible and biodegradable; mucoadhesive; enhances permeation; pH-sensitive release; modifiable for active targeting	Poor solubility at neutral pH; batch-to-batch variability; limited mechanical strength	[92]
	Alginate	Biocompatible; gel-forming; modifiable; good for protein/peptide encapsulation; supports multiple routes	Low mechanical stability without crosslinking; limited cell uptake without modifications	[93]
	Gelatin	Biodegradable; non-toxic; versatile for surface modification; good for protein drugs	Enzymatic degradation may be too rapid; requires crosslinking for stability	[94]
	PCL	Slow degradation—good for long-term release; high drug loading for hydrophobic drugs	Not ideal for rapid drug release; long-term persistence may cause accumulation	[95]
Targeting approach	Passive targeting (EPR effect)	Simple design; no need for complex ligand chemistry; good for solid tumors with leaky vasculature	Tumor heterogeneity limits EPR efficiency; significant off-target uptake (liver/spleen)	[96]
	Active targeting (ligands, antibodies, peptides)	Higher tumor specificity; receptor-mediated uptake; can bypass some resistance mechanisms	Requires detailed knowledge of tumor receptor profile; ligand conjugation adds complexity and cost; receptor heterogeneity may reduce efficacy	[97]
Delivery Routes	Intravenous (IV)	Enables systemic delivery; suitable for metastatic disease; can exploit both passive and active targeting	Risk of systemic toxicity; opsonization and clearance by MPS	[98]
	Pulmonary (inhalation)	Localized delivery to lung tumors; bypasses first-pass metabolism; reduces systemic toxicity	Formulation stability in aerosol form; variability in lung deposition; mucociliary clearance	[99]
	Oral	Patient compliance; potential for chronic therapy	Poor bioavailability for many drugs; degradation in GI tract; first-pass metabolism	[91]
	Intratumoral	High local concentration; minimal systemic exposure	Invasive; limited use for inaccessible tumors	[97]

**Table 4:** Imaging modalities integrated with polymeric nanoparticles for lung cancer theranostics.

Imaging Modality	Key Advantages and Limitations in Lung Cancer Theranostics	References
MRI	High soft-tissue contrast, non-ionizing; sensitive tracking of NP biodistribution; lower molecular sensitivity; gadolinium safety concerns.	[102]
CT	Excellent spatial resolution, widely available; uses ionizing radiation, low molecular sensitivity, possible metal-agent toxicity.	[103]
PET	Highest molecular sensitivity, quantitative biodistribution, detects metastases; short isotope half-lives, radiation exposure, complex radiochemistry.	[104]
SPECT	Good sensitivity, longer-lived isotopes, whole-body tracking; lower spatial resolution than PET, regulatory hurdles.	[94]
Optical (NIR, NIR-II)	High sensitivity, real-time surgical guidance; limited tissue penetration, autofluorescence, mainly preclinical use.	[105]
Ultrasound/Photoacoustic	Real-time, no ionizing radiation, depth improvement with PA; lung air limits access, microbubble stability issues.	[106]

**Table 5:** Studies for polymeric NPs for lung cancer management.

Type of Polymeric NPs	Drug Encapsulated/Cell Line Used	In Vitro/In Vivo Studies	Outcome	References
Chitosan	Carbamazepine	In vivo	Enhanced brain targeting and therapeutic efficacy at 219 nm and 80% drug entrapment	[92]
Glycol-modified chitosan	Hepatitis B vaccines	In vivo	Enhanced mucosal delivery	[108]
Chitosan	DOX	In vivo	Enhanced intestinal absorption	[137]
Chitosan	Paclitaxel—MDA-MB-231 breast cancer cells	Both	Enhanced anticancer activity compared to free paclitaxel in MDA-MB-231 breast cancer cells	[137]
Chitosan	DOX, siRNA, IGF-1R—A549 lung cancer cell line	In vitro	Improve anticancer efficacy in A549 lung cancer cell line	[110]
Chitosan	Berberine—urethane-induced lung cancer in male albino mice	In vivo	Prevented tumor angiogenesis by reducing levels of serum VEGFR2 and lung HIF-1 gene expression, oral administration and eco-friendly alternative	[112]
Gelatin	Nivolumab—A549 lung carcinoma cell	In vitro	87.88% inhibition rate, increased potency	[122]
PEGylated gelatin	Paclitaxel NSCLC	Both	Highest antiproliferative effect, 100% survival, maximum tumor inhibition	[94]
Gelatin	Concanavalin A—A549 lung cancer cells	In vitro	Increased apoptosis by boosted drug release	[123]
Gelatin	APS7 and cisplatin—A549 cells	In vitro	Improved cisplatin efficacy by blocking nicotine-induced calcium influx	[124]
Polycaprolactone	DOX—E0771 breast cancer cells and lung models	Both	Reduced DOX cardiotoxicity and improved anticancer activity	[131]
Poly(amidoamine)	Docetaxel (DTX)-alendronate (ALN)—A549 cells and mouse model	Both	Enhanced DTX anticancer activity Suppressed bone resorption, pain response, and growth of bone metastases	[135]

**Table 6:** Summary of clinical trials on polymeric NPs for lung cancer management.

Clinical Trial ID/Registration No.	Polymeric NP Type/Formulation	Drug/Therapeutic Agent	Study Phase	Study Design	Target Population	Primary Outcome Measures	Status/Sponsor	Reference
NCT00729612	Albumin based NP	Paclitaxel	Phase II	Interventional	NSCLC, Advanced Stage	PFS, OS	Completed/Greg Otterson, Ohio State University Comprehensive Cancer Center	[139]
NCT02240238	Cisplatin NP NC 6004	Gemcitabine	Phase I and II	Interventional	NSCLC, solid tumors	Safety, MTD	Completed/Nano Carrier Co., Ltd.	[140]
NCT00073723	Protein formulation	ABI-007, Protein formulation of paclitaxel	Phase I and II	Interventional	NSCLC	Safety, MTD	Completed/Celgene Corporation	[141]
NCT04314895	NanoPac (sterile nanoparticulate) intratumoral injection	Paclitaxel	Phase II	Interventional	NSCLC, SCLC, Neoplasm of lung	Safety, MTD	Completed/NanOlogy, LLC.	[140]
NCT02740985	Capsule nanoparticle suspension	Durvalumab AZD4635	Phase Ib	Interventional	NSCLC Advanced solid malignancies	PFS, ORR, Safety	Completed/AstraZeneca	[142]

**Table 7:** Factors influencing EPR effect variability and their impact on clinical translation.

Factor	Influence on EPR Effect	Impact on Clinical Translation	References
Tumor type	Highly angiogenic tumors (e.g., liver, some breast cancers) exhibit greater vascular permeability; desmoplastic tumors (e.g., pancreatic, some lung tumors) have restricted NP extravasation.	Nanomedicine accumulation varies widely; EPR-based delivery more effective in leaky tumors, less so in fibrotic or poorly vascularized tumors.	[147]
Tumor size	Small, rapidly growing tumors have immature, leaky vasculature; large tumors may have hypoxic or necrotic cores with poor perfusion.	Drug penetration is reduced in large tumors; dosing strategies may need to be tailored to tumor growth stage.	[148]
Tumor location	Location determines local blood flow and vessel permeability; tumors in highly vascularized organs differ from those in low-perfusion or protected areas (e.g., brain).	EPR effect is less pronounced in tumors with restricted access (e.g., behind the BBB), limiting passive targeting efficacy.	[149]
Tumor microenvironment	Dense extracellular matrix, high interstitial fluid pressure, and stromal barriers reduce NP penetration.	May necessitate combination strategies (e.g., stromal modulation, vascular normalization) to enhance delivery.	[147]
Patient-specific physiology	Variations in vascular density, permeability, and immune response affect NP clearance and distribution.	Personalized nanomedicine design and patient stratification may improve therapeutic predictability.	[148]

## Data Availability

No new data were created.

## References

[1] Allemani C., Matsuda T., Di Carlo V., Harewood R., Matz M., Nikšić M., Bonaventure A., Valkov M., Johnson C.J., Estève J. (2018). Global Surveillance of Trends in Cancer Survival 2000–14 (Concord-3): Analysis of Individual Records for 37,513,025 Patients Diagnosed with One of 18 Cancers from 322 Population-Based Registries in 71 Countries. Lancet.

[2] Sung H., Ferlay J., Siegel R.L., Laversanne M., Soerjomataram I., Jemal A., Bray F. (2021). Global Cancer Statistics 2020: Globocan Estimates of Incidence and Mortality Worldwide for 36 Cancers in 185 Countries. CA Cancer J. Clin..

[3] Malhotra J., Malvezzi M., Negri E., La Vecchia C., Boffetta P. (2016). Risk Factors for Lung Cancer Worldwide. Eur. Respir. J..

[4] Brinklov S., Kalko E.K.V., Surlykke A. (2009). Intense Echolocation Calls from Two ‘Whispering’ Bats, *Artibeus jamaicensis* and *Macrophyllum macrophyllum* (*Phyllostomidae*). J. Exp. Biol..

[5] Bade B.C., Cruz C.S.D. (2020). Lung Cancer 2020: Epidemiology, Etiology, and Prevention. Clin. Chest Med..

[6] Li Y., Wang N., Huang Y., He S., Bao M., Wen C., Wu L. (2024). Circmybl1 Suppressed Acquired Resistance to Osimertinib in Non-Small-Cell Lung Cancer. Cancer Genet..

[7] Wang S., Liu G., Yu L., Zhang C., Marcucci F., Jiang Y. (2024). Fluorofenidone Enhances Cisplatin Efficacy in Non-Small Cell Lung Cancer: A Novel Approach to Inhibiting Cancer Progression. Transl. Lung Cancer Res..

[8] Gao F., Yan G., Sun L., Xia H., Guo Z., Lin H., Du G. (2023). The Real Mechanisms of Emodin Treating Lung Cancer Based on System Pharmacology. Eurasian J. Med. Oncol..

[9] Wang J., Dong X., Liu Y., Lin K., Chen J. (2025). Copy Number Gain of Met Gene with Low Level in a Metastatic Lung Adenocarcinoma Patient Represents Response to Salvage Treatment with Savolitinib and Osimertinib: A Case Report. Front. Oncol..

[10] Ferlay J., Colombet M., Soerjomataram I., Parkin D.M., Piñeros M., Znaor A., Bray F. (2021). Cancer Statistics for the Year 2020: An Overview. Int. J. Cancer.

[11] Zhang Y., Liu C., Chen X., Zhang Y., Li Y., Hu X. (2024). Effects of Web-Based Acceptance and Commitment Therapy on Health-Related Outcomes among Patients with Lung Cancer: A Feasibility Randomized Controlled Trial. Psychooncology.

[12] El-Megharbel S.M., Albogami B., Hassoubah S.A., Beyari E.A., Albaqami N.M., Alsolami K., Hamza R.Z. (2024). Spectral Analysis of Novel Minocycline/Zn Complex with Promising Anticancer Activities against Large Lung Cancer Cells (H460), Antibacterial and Antioxidant Activities against Acrylamide-Induced Pulmonary Toxicity in Male Rats. Int. J. Pharmacol..

[13] Samet J.M., Avila-Tang E., Boffetta P., Hannan L.M., Olivo-Marston S., Thun M.J., Rudin C.M. (2009). Lung Cancer in Never Smokers: Clinical Epidemiology and Environmental Risk Factors. Clin. Cancer Res..

[14] Herbst R.S., Morgensztern D., Boshoff C. (2018). The Biology and Management of Non-Small Cell Lung Cancer. Nature.

[15] McGarry H.A. (2013). Mechanistic Links between Copd and Lung Cancer. Nat. Rev. Cancer.

[16] Skoulidis F., Heymach J.V. (2019). Co-Occurring Genomic Alterations In non-Small-Cell Lung Cancer Biology and Therapy. Nat. Rev. Cancer.

[17] George J., Lim J.S., Jang S.J., Cun Y., Ozretić L., Kong G., Leenders F., Lu X., Fernández-Cuesta L., Bosco G. (2015). Comprehensive Genomic Profiles of Small Cell Lung Cancer. Nature.

[18] Baylin S.B., Jones P.A. (2016). Epigenetic Determinants of Cancer. Cold Spring Harb. Perspect. Biol..

[19] New York State Department of Environmental Conservation, Division of Fish Wildlife and Marine Resources (2009). Guidelines for Conducting Bird and Bat Studies at Commercial Wind Energy Projects.

[20] Hinshaw D.C., Shevde L.A. (2019). The Tumor Microenvironment Innately Modulates Cancer Progression. Cancer Res..

[21] Arandhara A., Bhuyan P., Das B.K. (2025). Exploring Lung Cancer Microenvironment: Pathways and Nanoparticle-Based Therapies. Discov. Oncol..

[22] Zhang X., Zhang X., Yong T., Gan L., Yang X. (2024). Boosting Antitumor Efficacy of Nanoparticles by Modulating Tumor Mechanical Microenvironment. eBioMedicine.

[23] Sabit H., Pawlik T.M., Radwan F., Abdel-Hakeem M., Abdel-Ghany S., Wadan A.-H.S., Elzawahri M., El-Hashash A., Arneth B. (2025). Precision Nanomedicine: Navigating the Tumor Microenvironment for Enhanced Cancer Immunotherapy and Targeted Drug Delivery. Mol. Cancer.

[24] Mitchell M.J., Billingsley M.M., Haley R.M., Wechsler M.E., Peppas N.A., Langer R. (2021). Engineering Precision Nanoparticles for Drug Delivery. Nat. Rev. Drug Discov..

[25] Li L., Li J., Zhong M., Wu Z., Wan S., Li X., Zhang Y., Lv K. (2025). Nanozyme-Enhanced Tyramine Signal Amplification Probe for Preamplification-Free Myocarditis-Related Mirnas Detection. Chem. Eng. J..

[26] Feng C., Wang Y., Xu J., Zheng Y., Zhou W., Wang Y., Luo C. (2024). Precisely Tailoring Molecular Structure of Doxorubicin Prodrugs to Enable Stable Nanoassembly, Rapid Activation, Potent Antitumor Effect. Pharmaceutics.

[27] Gao Y., Wang Y., Jiang J., Wei P., Sun H. (2025). Triggered “on/Off” Luminescent Polypeptide Bowl-Shaped Nanoparticles for Selective Lighting of Tumor Cells. Small.

[28] Rani V., Venkatesan J., Prabhu A. (2022). Liposomes—A Promising Strategy for Drug Delivery in Anticancer Applications. J. Drug Deliv. Sci. Technol..

[29] Kumari P., Ghosh B., Biswas S. (2016). Nanocarriers for Cancer-Targeted Drug Delivery. J. Drug Target..

[30] Dong Q., Jiang Z. (2024). Platinum–Iron Nanoparticles for Oxygen-Enhanced Sonodynamic Tumor Cell Suppression. Inorganics.

[31] Mi P. (2020). Stimuli-Responsive Nanocarriers for Drug Delivery, Tumor Imaging, Therapy and Theranostics. Theranostics.

[32] Wang Y., Xu Y., Song J., Liu X., Liu S., Yang N., Wang L., Liu Y., Zhao Y., Zhou W. (2024). Tumor Cell-Targeting and Tumor Microenvironment-Responsive Nanoplatforms for the Multimodal Imaging-Guided Photodynamic/Photothermal/Chemodynamic Treatment of Cervical Cancer. Int. J. Nanomed..

[33] Madaan K., Kumar S., Poonia N., Lather V., Pandita D. (2014). Dendrimers in Drug Delivery and Targeting: Drug-Dendrimer Interactions and Toxicity Issues. J. Pharm. Bioallied Sci..

[34] Jafari S., Derakhshankhah H., Alaei L., Fattahi A., Varnamkhasti B.S., Saboury A.A. (2019). Mesoporous Silica Nanoparticles for Therapeutic/Diagnostic Applications. Biomed. Pharmacother..

[35] Aqil F., Gupta R.C. (2022). Exosomes in Cancer Therapy. Cancers.

[36] Anselmo A.C., Prabhakarpandian B., Pant K., Mitragotri S. (2017). Clinical and Commercial Translation of Advanced Polymeric Nanoparticle Systems: Opportunities and Material Challenges. Transl. Mater. Res..

[37] Gaspar R., Duncan R. (2009). Polymeric Carriers: Preclinical Safety and the Regulatory Implications for Design and Development of Polymer Therapeutics. Adv. Drug Deliv. Rev..

[38] Feng J., Zhang P., Wang D., Li Y., Tan J. (2024). New Strategies for Lung Cancer Diagnosis and Treatment: Applications and Advances in Nanotechnology. Biomark. Res..

[39] García-Fernández C., Fornaguera C., Borrós S. (2020). Nanomedicine in Non-Small Cell Lung Cancer: From Conventional Treatments to Immunotherapy. Cancers.

[40] Owen D.H., Wei L., Bertino E.M., Edd T., Villalona-Calero M.A., He K., Shields P.G., Carbone D.P., Otterson G.A. (2018). Incidence, Risk Factors and Effect on Survival of Immune-Related Adverse Events in Patients with Non-Small-Cell Lung Cancer. Clin. Lung Cancer.

[41] Chen D., Liu J., Wu J., Suk J.S. (2021). Enhancing Nanoparticle Penetration through Airway Mucus to Improve Drug Delivery Efficacy in the Lung. Expert Opin. Drug Deliv..

[42] Sarma K., Akther M.H., Ahmad I., Afzal O., Altamimi A.S.A., Alossaimi M.A., Jaremko M., Emwas A.-H., Gautam P. (2024). Adjuvant Novel Nanocarrier-Based Targeted Therapy for Lung Cancer. Molecules.

[43] Antonia S.J., Villegas A., Daniel D., Vicente D., Murakami S., Hui R., Yokoi T., Chiappori A., Lee K.H., de Wit M. (2017). Durvalumab after Chemoradiotherapy in Stage Iii Non-Small-Cell Lung Cancer. N. Engl. J. Med..

[44] Hellmann M.D., Ciuleanu T.E., Pluzanski A., Lee J.S., Otterson G.A., Audigier-Valette C., Minenza E., Linardou H., Burgers S., Salman P. (2018). Nivolumab Plus Ipilimumab in Lung Cancer with a High Tumor Mutational Burden. N. Engl. J. Med..

[45] Soria J.C., Ohe Y., Vansteenkiste J., Reungwetwattana T., Chewaskulyong B., Lee K.H., Dechaphunkul A., Imamura F., Nogami N., Kurata T. (2018). Osimertinib in Untreated Egfr-Mutated Advanced Non-Small-Cell Lung Cancer. N. Engl. J. Med..

[46] Rittmeyer A., Barlesi F., Waterkamp D., Park K., Ciardiello F., von Pawel J., Gadgeel S.M., Hida T., Kowalski D.M., Dols M.C. (2017). Atezolizumab Versus Docetaxel in Patients with Previously Treated Non-Small-Cell Lung Cancer (Oak): A Phase 3, Open-Label, Multicentre Randomised Controlled Trial. Lancet.

[47] (2018). Gourd, Alectinib Shows Cns Efficacy in Alk-Positive Nsclc. Lancet Oncol..

[48] Ando K., Manabe R., Kishino Y., Kusumoto S., Yamaoka T., Tanaka A., Ohmori T., Sagara H. (2023). Comparative Efficacy of Alk Inhibitors for Treatment-Naïve Alk-Positive Advanced Non-Small Cell Lung Cancer with Central Nervous System Metastasis: A Network Meta-Analysis. Int. J. Mol. Sci..

[49] Lindeman N.I., Cagle P.T., Aisner D.L., Arcila M.E., Beasley M.B., Bernicker E.H., Colasacco C., Dacic S., Hirsch F.R., Kerr K. (2018). Updated Molecular Testing Guideline for the Selection of Lung Cancer Patients for Treatment with Targeted Tyrosine Kinase Inhibitors: Guideline from the College of American Pathologists, the International Association for the Study of Lung Cancer, and the Association for Molecular Pathology. Arch. Pathol. Lab. Med..

[50] Rolfo C., Mack P., Scagliotti G.V., Aggarwal C., Arcila M.E., Barlesi F., Bivona T., Diehn M., Dive C., Dziadziuszko R. (2021). Liquid Biopsy for Advanced Nsclc: A Consensus Statement from the International Association for the Study of Lung Cancer. J. Thorac. Oncol..

[51] Schilsky R.L. (2010). Personalized Medicine in Oncology: The Future Is Now. Nat. Rev. Drug Discov..

[52] Pascual J., Attard G., Bidard F.C., Curigliano G., De Mattos-Arruda L., Diehn M., Italiano A., Lindberg J., Merker J.D., Montagut C. (2022). Esmo Recommendations on the Use of Circulating Tumour DNA Assays for Patients with Cancer: A Report from the Esmo Precision Medicine Working Group. Ann. Oncol..

[53] Tsuboi M., Herbst R.S., John T., Kato T., Majem M., Grohé C., Wang J., Goldman J.W., Lu S., Su W.C. (2023). Overall Survival with Osimertinib in Resected Egfr-Mutated Nsclc. N. Engl. J. Med..

[54] Reck M., Remon J., Hellmann M.D. (2022). First-Line Immunotherapy for Non-Small-Cell Lung Cancer. J. Clin. Oncol..

[55] Ramalingam S.S., Vansteenkiste J., Planchard D., Cho B.C., Gray J.E., Ohe Y., Zhou C., Reungwetwattana T., Cheng Y., Chewaskulyong B. (2020). Overall Survival with Osimertinib in Untreated, Egfr-Mutated Advanced Nsclc. N. Engl. J. Med..

[56] Kievit F.M., Zhang M. (2011). Cancer Nanotheranostics: Improving Imaging and Therapy by Targeted Delivery across Biological Barriers. Adv. Mater..

[57] Wahajuddin, Arora S. (2012). Superparamagnetic Iron Oxide Nanoparticles: Magnetic Nanoplatforms as Drug Carriers. Int. J. Nanomed..

[58] Manescu V., Antoniac I., Paltanea G., Nemoianu I.V., Mohan A.G., Antoniac A., Rau J.V., Laptoiu S.A., Mihai P., Gavrila H. (2024). Magnetic Hyperthermia in Glioblastoma Multiforme Treatment. Int. J. Mol. Sci..

[59] Mykhaylyk O., Sanchez-Antequera Y., Vlaskou D., Cerda M.B., Bokharaei M., Hammerschmid E., Anton M., Plank C. (2015). Magnetic Nanoparticle and Magnetic Field Assisted Sirna Delivery in Vitro. Methods Mol. Biol..

[60] Wei X., Song M., Li W., Huang J., Yang G., Wang Y. (2021). Multifunctional Nanoplatforms Co-Delivering Combinatorial Dual-Drug for Eliminating Cancer Multidrug Resistance. Theranostics.

[61] Li X., Younis M.H., Wei W., Cai W. (2023). Pd-L1 - Targeted Magnetic Fluorescent Hybrid Nanoparticles: Illuminating the Path of Image-Guided Cancer Immunotherapy. Eur. J. Nucl. Med. Mol. Imaging.

[62] Arami H., Khandhar A., Liggitt D., Krishnan K.M., Delivery I.V. (2015). Pharmacokinetics, Biodistribution and Toxicity of Iron Oxide Nanoparticles. Chem. Soc. Rev..

[63] Müller R.H., Mäder K., Gohla S. (2000). Solid Lipid Nanoparticles (Sln) for Controlled Drug Delivery—A Review of the State of the Art. Eur. J. Pharm. Biopharm..

[64] Sivadasan D., Ramakrishnan K., Mahendran J., Ranganathan H., Karuppaiah A., Rahman H. (2023). Solid Lipid Nanoparticles: Applications and Prospects in Cancer Treatment. Int. J. Mol. Sci..

[65] Song Y., Cai H., Yin T., Huo M., Ma P., Zhou J., Lai W. (2018). Paclitaxel-Loaded Redox-Sensitive Nanoparticles Based on Hyaluronic Acid-Vitamin E Succinate Conjugates for Improved Lung Cancer Treatment. Int. J. Nanomed..

[66] Chai Z., Ran D., Lu L., Zhan C., Ruan H., Hu X., Xie C., Jiang K., Li J., Zhou J. (2019). Ligand-Modified Cell Membrane Enables the Targeted Delivery of Drug Nanocrystals to Glioma. ACS Nano.

[67] Subhan M.A., Filipczak N., Torchilin V.P. (2023). Advances with Lipid-Based Nanosystems for Sirna Delivery to Breast Cancers. Pharmaceuticals.

[68] Al Khatib A.O., El-Tanani M., Al-Obaidi H. (2023). Inhaled Medicines for Targeting Non-Small Cell Lung Cancer. Pharmaceutics.

[69] Pechyen C., Tangnorawich B., Toommee S., Marks R., Parcharoen Y. (2024). Green Synthesis of Metal Nanoparticles, Characterization, and Biosensing Applications. Sens. Int..

[70] Tinajero-Díaz E., Salado-Leza D., Gonzalez C., Velázquez M.M., López Z., Bravo-Madrigal J., Knauth P., Flores-Hernández F.Y., Herrera-Rodríguez S.E., Navarro R.E. (2021). Green Metallic Nanoparticles for Cancer Therapy: Evaluation Models and Cancer Applications. Pharmaceutics.

[71] Abdel-Aziz M.M., Elella M.H.A., Mohamed R.R. (2020). Green Synthesis of Quaternized Chitosan/Silver Nanocomposites for Targeting Mycobacterium Tuberculosis and Lung Carcinoma Cells (a-549). Int. J. Biol. Macromol..

[72] Yang S.J., Lin F.H., Tsai K.C., Wei M.F., Tsai H.M., Wong J.M., Shieh M.J. (2010). Folic Acid-Conjugated Chitosan Nanoparticles Enhanced Protoporphyrin Ix Accumulation in Colorectal Cancer Cells. Bioconjug. Chem..

[73] Huang M., Myers C.R., Wang Y., You M. (2021). Mitochondria as a Novel Target for Cancer Chemoprevention: Emergence of Mitochondrial-Targeting Agents. Cancer Prev. Res..

[74] Baghani L., Heris N.N., Khonsari F., Dinarvand S., Dinarvand M., Atyabi F. (2022). Trimethyl-Chitosan Coated Gold Nanoparticles Enhance Delivery, Cellular Uptake and Gene Silencing Effect of Egfr-Sirna in Breast Cancer Cells. Front. Mol. Biosci..

[75] Kulkarni-Dwivedi N., Patel P.R., Shravage B.V., Umrani R.D., Paknikar K.M., Jadhav S.H. (2022). Hyperthermia and Doxorubicin Release by Fol-Lsmo Nanoparticles Induce Apoptosis and Autophagy in Breast Cancer Cells. Nanomedicine.

[76] Quesada-González D., Merkoçi A. (2025). Quantum Dots for Biosensing: Classification and Applications. Biosens. Bioelectron..

[77] Pareek A., Kumar D., Pareek A., Gupta M.M. (2025). Advancing Cancer Therapy with Quantum Dots and Other Nanostructures: A Review of Drug Delivery Innovations, Applications, and Challenges. Cancers.

[78] Ren L., Wang L., Rehberg M., Stoeger T., Zhang J., Chen S. (2021). Applications and Immunological Effects of Quantum Dots on Respiratory System. Front. Immunol..

[79] Emami F., Duwa R., Banstola A., Woo S.M., Kwon T.K., Yook S. (2023). Dual Receptor Specific Nanoparticles Targeting Egfr and Pd-L1 for Enhanced Delivery of Docetaxel in Cancer Therapy. Biomed. Pharmacother..

[80] Yi Y., Zhao H. (2025). Revolutionizing Tissue Clearing and 3-Dimensional Imaging: Transparent Embedding Solvent System for Uniform High-Resolution Imaging. BME Front..

[81] Merlin J.P.J., Crous A. (2024). Abrahamse, Combining Photodynamic Therapy and Targeted Drug Delivery Systems: Enhancing Mitochondrial Toxicity for Improved Cancer Outcomes. Int. J. Mol. Sci..

[82] Mohkam M., Sadraeian M., Lauto A., Gholami A., Nabavizadeh S.H., Esmaeilzadeh H., Alyasin S. (2023). Exploring the Potential and Safety of Quantum Dots in Allergy Diagnostics. Microsyst. Nanoeng..

[83] Getz T., Qin J., Medintz I.L., Delehanty J.B., Susumu K., Dawson P.E., Dawson G. (2016). Quantum Dot-Mediated Delivery of Sirna to Inhibit Sphingomyelinase Activities in Brain-Derived Cells. J. Neurochem..

[84] Woutersen M., Muller A., Pronk M.E.J., Cnubben N.H.P., Hakkert B.C. (2020). Regulating Human Safety: How Dose Selection in Toxicity Studies Impacts Human Health Hazard Assessment and Subsequent Risk Management Options. Regul. Toxicol. Pharmacol..

[85] Jin M., Hou Y., Quan X., Chen L., Gao Z., Huang W. (2021). Smart Polymeric Nanoparticles with Ph-Responsive and Peg-Detachable Properties (Ii): Co-Delivery of Paclitaxel and Vegf Sirna for Synergistic Breast Cancer Therapy in Mice. Int. J. Nanomed..

[86] Tiwari R., Patil A., Verma R., Deva V., Rudrangi S.R.S., Bhise M.R., Vinukonda A. (2025). Biofunctionalized Polymeric Nanoparticles for the Enhanced Delivery of Erlotinib in Cancer Therapy. J. Biomater. Sci. Polym. Ed..

[87] Beach M.A., Nayanathara U., Gao Y., Zhang C., Xiong Y., Wang Y., Such G.K. (2024). Polymeric Nanoparticles for Drug Delivery. Chem. Rev..

[88] Thambiliyagodage C., Jayanetti M., Mendis A., Ekanayake G., Liyanaarachchi H., Vigneswaran S. (2023). Recent Advances in Chitosan-Based Applications—A Review. Materials.

[89] Sharfi L., Nowroozi M.R., Smirnova G., Fedotova A., Babarykin D., Mirshafiey A. (2024). The Safety Properties of Sodium Alginate and Its Derivatives. Br. J. Healthc. Med. Res..

[90] Mahar R., Chakraborty A., Nainwal N., Bahuguna R., Sajwan M., Jakhmola V. (2023). Application of Plga as a Biodegradable and Biocompatible Polymer for Pulmonary Delivery of Drugs. AAPS PharmSciTech.

[91] Moharir K., Kale V., Ittadwar A., Paul M.K. (2021). Polymeric Nanoparticle-Based Drug–Gene Delivery for Lung Cancer. Handbook of Lung Targeted Drug Delivery Systems.

[92] Kuen C.Y., Masarudin M.J. (2022). Chitosan Nanoparticle-Based System: A New Insight into the Promising Controlled Release System for Lung Cancer Treatment. Molecules.

[93] Yasamineh S., Gholizadeh O., Kalajahi H.G., Yasamineh P., Firouzi-Amandi A., Dadashpour M. (2023). Future Prospects of Natural Polymer-Based Drug Delivery Systems in Combating Lung Diseases. Natural Polymeric Materials Based Drug Delivery Systems in Lung Diseases.

[94] Gu M., Luan J., Song K., Qiu C., Zhang X., Zhang M. (2021). Development of Paclitaxel Loaded Pegylated Gelatin Targeted Nanoparticles for Improved Treatment Efficacy in Non-Small Cell Lung Cancer (Nsclc): An in Vitro and in Vivo Evaluation Study. Acta Biochim. Pol..

[95] Dhuri A., Sriram A., Aalhate M., Mahajan S., Parida K.K., Singh H., Gupta U., Maji I., Guru S.K., Singh P.K. (2023). Chitosan Functionalized Pcl Nanoparticles Bearing Tyrosine Kinase Inhibitor Osimertinib Mesylate for Effective Lung Cancer Therapy. Pharm. Dev. Technol..

[96] Vagena I.-A., Malapani C., Gatou M.-A., Lagopati N., Pavlatou E.A. (2025). Enhancement of Epr Effect for Passive Tumor Targeting: Current Status and Future Perspectives. Appl. Sci..

[97] Sharma A., Shambhwani D., Pandey S., Singh J., Lalhlenmawia H., Kumarasamy M., Singh S.K., Chellappan D.K., Gupta G., Prasher P. (2022). Advances in Lung Cancer Treatment Using Nanomedicines. ACS Omega.

[98] Hani U., Begum M.Y., Wahab S., Siddiqua A. (2022). Riyaz Ali M Osmani, Mohamed Rahamathulla, A Comprehensive Review of Current Perspectives on Novel Drug Delivery Systems and Approaches for Lung Cancer Management. J. Pharm. Innov..

[99] Cojocaru E., Petriș O.R., Cojocaru C. (2024). Nanoparticle-Based Drug Delivery Systems in Inhaled Therapy: Improving Respiratory Medicine. Pharmaceuticals.

[100] Dahlsgaard-Wallenius S.E., Hildebrandt M.G., Johansen A., Vilstrup M.H., Petersen H., Gerke O., Høilund-Carlsen P.F., Morsing A., Andersen T.L. (2021). Hybrid Pet/Mri in Non-Small Cell Lung Cancer (Nsclc) and Lung Nodules—A Literature Review. Eur. J. Nucl. Med. Mol. Imaging.

[101] Batouty N.M., Saleh G.A., Sharafeldeen A., Kandil H., Mahmoud A., Shalaby A., Yaghi M., Khelifi A., Ghazal M., El-Baz A. (2022). State of the Art: Lung Cancer Staging Using Updated Imaging Modalities. Bioengineering.

[102] Anani T., Rahmati S., Sultana N., David A.E. (2021). Mri-Traceable Theranostic Nanoparticles for Targeted Cancer Treatment. Theranostics.

[103] Najdian A., Beiki D., Abbasi M., Gholamrezanezhad A., Ahmadzadehfar H., Amani A.M., Ardestani M.S., Assadi M. (2024). Exploring Innovative Strides in Radiolabeled Nanoparticle Progress for Multimodality Cancer Imaging and Theranostic Applications. Cancer Imaging.

[104] Freitas L.F., Ferreira A.H., Thipe V.C., Varca G.H.C., Lima C.S.A., Batista J.G.S., Riello F.N., Nogueira K., Cruz C.P.C., Mendes G.O.A. (2021). The State of the Art of Theranostic Nanomaterials for Lung, Breast, and Prostate Cancers. Nanomaterials.

[105] Zhao M., Leggett E., Bourke S., Poursanidou S., Carter-Searjeant S., Po S., Carmo M.P.D., Dailey L.A., Manning P., Ryan S.G. (2021). Theranostic near-Infrared-Active Conjugated Polymer Nanoparticles. ACS Nano.

[106] Wen Y., Guo D., Zhang J., Liu X., Liu T., Li L., Jiang S., Wu D., Jiang H. (2022). Clinical Photoacoustic/Ultrasound Dual-Modal Imaging: Current Status and Future Trends. Front. Physiol..

[107] Shaikh K.I.M.A.J., Afzal O., Altamimi A.S.A., Almalki W.H., Alzarea S.I., Al-Abbasi F.A., Pandey M., Dureja H., Singh S.K., Dua K. (2023). Chitosan-Based Nano Drug Delivery System for Lung Cancer. J. Drug Deliv. Sci. Technol..

[108] Pawar D., Jaganathan K.S. (2016). Mucoadhesive Glycol Chitosan Nanoparticles for Intranasal Delivery of Hepatitis B Vaccine: Enhancement of Mucosal and Systemic Immune Response. Drug Deliv..

[109] Zare M., Samani S.M., Sobhani Z. (2018). Enhanced Intestinal Permeation of Doxorubicin Using Chitosan Nanoparticles. Adv. Pharm. Bull..

[110] Shali H., Shabani M., Pourgholi F., Hajivalili M., Aghebati-Maleki L., Jadidi-Niaragh F., Baradaran B., Akbari A.A.M.-S., Younesi V., Yousefi M. (2018). Co-Delivery of Insulin-Like Growth Factor 1 Receptor Specific Sirna and Doxorubicin Using Chitosan-Based Nanoparticles Enhanced Anticancer Efficacy in A549 Lung Cancer Cell Line. Artif. Cells Nanomed. Biotechnol..

[111] Mahmood R.I., Al-Taie A., Al-Rahim A.M., Mohammed-Salih H.S., Ibrahim H.A., Albukhaty S., Jawad S.F., Jabir M.S., Salem M.M., Bekhit M.M. (2025). Biogenic Synthesized Selenium Nanoparticles Combined Chitosan Nanoparticles Controlled Lung Cancer Growth Via Ros Generation and Mitochondrial Damage Pathway. Nanotechnol. Rev..

[112] Mahmoud M.A., El-Bana M.A., Morsy S.M., Badawy E.A., Farrag A.-E., Badawy A.M., Abdel-Wahhab M.A., El-Dosoky M.A. (2022). Synthesis and Characterization of Berberine-Loaded Chitosan Nanoparticles for the Protection of Urethane-Induced Lung Cancer. Int. J. Pharm..

[113] Zhu X., Yu Z., Feng L., Deng L., Fang Z., Liu Z., Li Y., Wu X., Qin L., Guo R. (2021). Chitosan-Based Nanoparticle Co-Delivery of Docetaxel and Curcumin Ameliorates Anti-Tumor Chemoimmunotherapy in Lung Cancer. Carbohydr. Polym..

[114] Patel P., Raval M., Airao V., Ali N., Shazly G.A., Khan R., Prajapati B. (2024). Formulation of Folate Receptor-Targeted Silibinin-Loaded Inhalable Chitosan Nanoparticles by the Qbd Approach for Lung Cancer Targeted Delivery. ACS Omega.

[115] Rostami E. (2022). Recent Achievements in Sodium Alginate-Based Nanoparticles for Targeted Drug Delivery. Polym. Bull..

[116] Huang J., Guo J., Zhu J., Zou X. (2022). Supported Silver Nanoparticles over Alginate-Modified Magnetic Nanoparticles: Synthesis, Characterization and Treat the Human Lung Carcinoma. J. Saudi Chem. Soc..

[117] Veysi A., Yaghoobi-Ershadi M.R., Rassi Y., Hosseini-Vasoukolaei N., Jeddi-Tehrani M., Rezaee-Node A., Gholampour F., Saeidi Z., Fatemi M., Arandian M.H. (2017). Rearing and Biology of Phlebotomus Sergenti, the Main Vector of Anthroponotic Cutaneous Leishmaniasis in Iran. J. Arthropod-Borne Dis..

[118] Işıklan N., Geyik G., Güncüm E. (2024). Alginate-Based Bio-Nanocomposite Reinforced with Poly(2-Hydroxypropyl Methacrylamide) and Magnetite Graphene Oxide for Delivery of Etoposide and Photothermal Therapy. Mater. Today Chem..

[119] Parashar A.K., Saraogi G.K., Tiwari B.K., Tyagi L.K., Sethi V.A., Shrivastava V., Sharma R., Pandey V., Mishra N. (2024). Polymeric Nanoparticles-Based Strategies for Cancer Immunotherapy. Nanotechnology Based Strategies for Cancer Immunotherapy: Concepts, Design, and Clinical Applications.

[120] Milano F., Masi A., Madaghiele M., Sannino A., Salvatore L., Gallo N. (2023). Current Trends in Gelatin-Based Drug Delivery Systems. Pharmaceutics.

[121] Wu D., Cao X.-H., Jia P.-Z., Zeng Y.-J., Feng Y.-X., Tang L.-M., Zhou W.-X., Chen K.-Q. (2020). Excellent Thermoelectric Performance in Weak-Coupling Molecular Junctions with Electrode Doping and Electrochemical Gating. Sci. China Phys. Mech. Astron..

[122] Ali D.S., Gad H.A., Hathout R.M. (2024). Enhancing Effector Jurkat Cell Activity and Increasing Cytotoxicity against A549 Cells Using Nivolumab as an Anti-Pd-1 Agent Loaded on Gelatin Nanoparticles. Gels.

[123] Vaghasiya K., Ray E., Singh R., Jadhav K., Sharma A., Khan R., Katare O.P., Verma R.K. (2021). Efficient, Enzyme Responsive and Tumor Receptor Targeting Gelatin Nanoparticles Decorated with Concanavalin-a for Site-Specific and Controlled Drug Delivery for Cancer Therapy. Mater. Sci. Eng. C.

[124] Kononenko V., Joukhan A., Bele T., Križaj I., Kralj S., Turk T., Drobne D. (2024). Gelatin Nanoparticles Loaded with 3-Alkylpyridinium Salt Aps7, an Analog of Marine Toxin, Are a Promising Support in Human Lung Cancer Therapy. Biomed. Pharmacother..

[125] Chen Y.-J., Wang Z.-W., Lu T.-L., Gomez C.B., Fang H.-W., Wei Y., Tseng C.-L. (2020). The Synergistic Anticancer Effect of Dual Drug-(Cisplatin/Epigallocatechin Gallate) Loaded Gelatin Nanoparticles for Lung Cancer Treatment. J. Nanomater..

[126] Mehrotra N., Anees M., Tiwari S., Kharbanda S., Singh H. (2023). Polylactic Acid Based Polymeric Nanoparticle Mediated Co-Delivery of Navitoclax and Decitabine for Cancer Therapy. Nanomedicine.

[127] Wang X., Choudhary S.M., Chauhan G., Muth A., Gupta V. (2025). Transferrin-Conjugated Polymeric Nanoparticles for Receptor-Mediated Delivery of Resveratrol-Cyclodextrin Complex in Non-Small Cell Lung Cancer (Nsclc) Cells. J. Drug Deliv. Sci. Technol..

[128] Yao F., Lin L., Shi W., Li C., Liang Z., Huang C. (2022). Inhibitory Effect of Poly(Lactic-Co-Glycolic Acid) Nanoparticles Loaded with Resveratrol and Phosphatase and Tensin Homolog Deleted on Chromosome Ten (Pten) Sirna on Lung Cancer Cells. Sci. Adv. Mater..

[129] Niza E., Ocaña A., Castro-Osma J.A., Bravo I., Alonso-Moreno C. (2021). Polyester Polymeric Nanoparticles as Platforms in the Development of Novel Nanomedicines for Cancer Treatment. Cancers.

[130] Lombardo R., Ruponen M., Rautio J., Lampinen R., Kanninen K.M., Koivisto A.M., Penttilä E., Löppönen H., Demartis S., Giunchedi P. (2024). A Technological Comparison of Freeze-Dried Poly-ɛ-Caprolactone (Pcl) and Poly (Lactic-Co-Glycolic Acid) (Plga) Nanoparticles Loaded with Clozapine for Nose-to-Brain Delivery. J. Drug Deliv. Sci. Technol..

[131] Cabeza L., Ortiz R., Prados J., Delgado Á.V., Martín-Villena M.J., Clares B., Perazzoli G., Entrena J.M., Melguizo C., Arias J.L. (2017). Improved Antitumor Activity and Reduced Toxicity of Doxorubicin Encapsulated in Poly(ε-Caprolactone) Nanoparticles in Lung and Breast Cancer Treatment: An in Vitro and in Vivo Study. Eur. J. Pharm. Sci..

[132] Akbari E., Mousazadeh H., Hanifehpour Y., Mostafavi E., Gorabi A.M., Nejati K., Peyman keyhanvar, Pazoki-Toroudi H., Mohammadhosseini M., Akbarzadeh A. (2022). Co-Loading of Cisplatin and Methotrexate in Nanoparticle-Based Pcl-Peg System Enhances Lung Cancer Chemotherapy Effects. J. Clust. Sci..

[133] Aorada S., Chittasupho C., Mangmool S., Angerhofer A., Imaram W. (2024). Gallic Acid-Encapsulated Pamam Dendrimers as an Antioxidant Delivery System for Controlled Release and Reduced Cytotoxicity against Arpe-19 Cells. Bioconjug. Chem..

[134] Guo Z., Li S., Liu Z., Xue W. (2018). Tumor-Penetrating Peptide-Functionalized Redox-Responsive Hyperbranched Poly(Amido Amine) Delivering Sirna for Lung Cancer Therapy. ACS Biomater. Sci. Eng..

[135] Bai S.-B., Cheng Y., Liu D.-Z., Ji Q.-F., Liu M., Zhang B.-L., Mei Q.-B., Zhou S.-Y. (2020). Bone-Targeted Pamam Nanoparticle to Treat Bone Metastases of Lung Cancer. Nanomedicine.

[136] Yin Y., Li Y., Wang S., Dong Z., Liang C., Sun J., Wang C., Chai R., Fei W., Zhang J. (2021). Mscs-Engineered Biomimetic Pmaa Nanomedicines for Multiple Bioimaging-Guided and Photothermal-Enhanced Radiotherapy of Nsclc. J. Nanobiotechnol..

[137] Zare H., Ahmadi S., Ghasemi A., Ghanbari M., Rabiee N., Bagherzadeh M., Karimi M., Webster T.J., Hamblin M.R., Mostafavi E. (2021). Carbon Nanotubes: Smart Drug/Gene Delivery Carriers. Int. J. Nanomed..

[138] Hanafy N.A.N. (2025). Optimally Designed Pegylatied Arabinoxylan Paclitaxel Nano-Micelles as Alternative Delivery for Abraxane®: A Potential Targeted Therapy against Breast and Lung Cancers. Int. J. Biol. Macromol..

[139] Bertino E.M., Williams T.M., Shilo K., Villalona-Calero M.A., Phillips G.S., Mo X., Otterson G.A. (2014). Phase 2 Trial of Nab-Paclitaxel Plus Carboplatin for Advanced Nsclc in Patients at Risk of Bleeding from Vegf Directed Therapies: Metastatic Non-Small Cell Lung Cancer. Int. J. Radiat. Oncol. Biol. Phys..

[140] Subbiah V., Grilley-Olson J.E., Combest A.J., Sharma N., Tran R.H., Bobe I., Osada A., Takahashi K., Balkissoon J., Camp A. (2018). Phase Ib/Ii Trial of Nc-6004 (Nanoparticle Cisplatin) Plus Gemcitabine in Patients with Advanced Solid Tumors. Clin. Cancer Res..

[141] Ibrahim N.K., Desai N., Legha S., Soon-Shiong P., Theriault R.L., Rivera E., Esmaeli B., Ring S.E., Bedikian A., Hortobagyi G.N. (2002). Phase I and Pharmacokinetic Study of Abi-007, a Cremophor-Free, Protein-Stabilized, Nanoparticle Formulation of Paclitaxel. Clin. Cancer Res..

[142] Lim E.A., Bendell J.C., Falchook G.S., Bauer T.M., Drake C.G., Choe J.H., George D.J., Karlix J.L., Ulahannan S., Sachsenmeier K.F. (2022). Phase Ia/B, Open-LabelOpen-Label, Multicenter Study of Azd4635 (an Adenosine A2a Receptor Antagonist) as Monotherapy or Combined with Durvalumab, in Patients with Solid Tumors. Clin. Cancer Res..

[143] Ashique S., Garg A., Mishra N., Raina N., Ming L.C., Tulli H.S., Behl T., Rani R., Gupta M. (2023). Nano-Mediated Strategy for Targeting and Treatment of Non-Small Cell Lung Cancer (Nsclc). Naunyn-Schmiedeberg’s Arch. Pharmacol..

[144] Su P., Pei W., Wang X., Ma Y., Jiang Q., Liang J., Zhou S., Zhao J., Liu J., Lu G.Q.M. (2021). Exceptional Electrochemical Her Performance with Enhanced Electron Transfer between Ru Nanoparticles and Single Atoms Dispersed on a Carbon Substrate. Angew. Chem. Int. Ed. Engl..

[145] Srinivasarao D.A., Shah S., Famta P., Vambhurkar G., Jain N., Pindiprolu S., Sharma A., Kumar R., Padhy H.P., Kumari M. (2025). Unravelling the Role of Tumor Microenvironment Responsive Nanobiomaterials in Spatiotemporal Controlled Drug Delivery for Lung Cancer Therapy. Drug Deliv. Transl. Res..

[146] Xu W., Yang S., Lu L., Xu Q., Wu S., Zhou J., Lu J., Fan X., Meng N., Ding Y. (2023). Influence of Lung Cancer Model Characteristics on Tumor Targeting Behavior of Nanodrugs. J. Control. Release.

[147] Islam R., Maeda H., Fang J. (2022). Factors Affecting the Dynamics and Heterogeneity of the Epr Effect: Pathophysiological and Pathoanatomic Features, Drug Formulations and Physicochemical Factors. Expert. Opin. Drug Deliv..

[148] Subhan M.A. (2021). Satya Siva Kishan Yalamarty, Nina Filipczak, Farzana Parveen, Vladimir P Torchilin, Recent Advances in Tumor Targeting Via Epr Effect for Cancer Treatment. J. Pers. Med..

[149] Kim J., Cho H., Lim D.-K., Joo M.K., Kim K. (2023). Perspectives for Improving the Tumor Targeting of Nanomedicine Via the Epr Effect in Clinical Tumors. Int. J. Mol. Sci..

[150] Elmowafy M., Shalaby K., Elkomy M.H., Alsaidan O.A., Gomaa H.A.M., Abdelgawad M.A., Mostafa E.M. (2023). Polymeric Nanoparticles for Delivery of Natural Bioactive Agents: Recent Advances and Challenges. Polymers.

[151] Sabir F., Qindeel M., Zeeshan M., Ain Q.U., Rahdar A., Barani M., González E., Aboudzadeh M.A. (2021). Onco-Receptors Targeting in Lung Cancer Via Application of Surface-Modified and Hybrid Nanoparticles: A Cross-Disciplinary Review. Processes.

[152] Yu M., Mathew A., Liu D., Chen Y., Wu J., Zhang Y., Zhang N., Lamprou D.A., Weaver E. (2024). Microfluidics for Formulation and Scale-up Production of Nanoparticles for Biopharma Industry. Microfluidics in Pharmaceutical Sciences: Formulation, Drug Delivery, Screening, and Diagnostics.

[153] Worku D.A., Hewitt V. (2020). The Role and Economics of Immunotherapy in Solid Tumour Management. J. Oncol. Pharm. Pract..

[154] Cangut B., Akinlusi R., Mohseny A., Ghesani N., Ghesani M. (2025). Evolving Paradigms in Lung Cancer: Latest Trends in Diagnosis, Management, and Radiopharmaceuticals. Semin. Nucl. Med..

[155] Salaudeen H.D., Akinniranye R.D. (2024). Precision Nanotechnology for Early Cancer Detection and Biomarker Identification. Int. J. Res. Publ. Rev..

[156] Desai N., Rana D., Patel M., Bajwa N., Prasad R., Vora L.K. (2025). Nanoparticle Therapeutics in Clinical Perspective: Classification, Marketed Products, and Regulatory Landscape. Small.

[157] Junyaprasert V.B., Thummarati P. (2023). Innovative Design of Targeted Nanoparticles: Polymer–Drug Conjugates for Enhanced Cancer Therapy. Pharmaceutics.

[158] Yang T., Zhai J., Hu D., Yang R., Wang G., Li Y., Liang G. (2022). “Targeting Design” of Nanoparticles in Tumor Therapy. Pharmaceutics.

[159] Kim Y., Kim J., Eom S., Jun H., Lee H.B., Jeong D., Kang S. (2025). Protein Nanoparticles Simultaneously Displaying Trail and Egfr-Binding Ligands Effectively Induce Apoptotic Cancer Cell Death and Overcome Egfr-Tki Resistance in Lung Cancer. ACS Appl. Mater. Interfaces.

[160] Nagime P.V., Singh S., Chidrawar V.R., Rajput A., Syukri D.M., Marwan N.T., Shafi S. (2024). Moringa Oleifera: A Plethora of Bioactive Reservoirs with Tremendous Opportunity for Green Synthesis of Silver Nanoparticles Enabled with Multifaceted Applications. Nano-Struct. Nano-Objects.

[161] Rakaee M., Tafavvoghi M., Ricciuti B., Alessi J.V., Cortellini A., Citarella F., Nibid L., Perrone G., Adib E., Fulgenzi C.A.M. (2025). Deep Learning Model for Predicting Immunotherapy Response in Advanced Non-Small Cell Lung Cancer. JAMA Oncol..

